# Toward Tailoring Just-in-Time Adaptive Intervention Systems for Workplace Stress Reduction: Exploratory Analysis of Intervention Implementation

**DOI:** 10.2196/48974

**Published:** 2024-09-12

**Authors:** Jina Suh, Esther Howe, Robert Lewis, Javier Hernandez, Koustuv Saha, Tim Althoff, Mary Czerwinski

**Affiliations:** 1 Microsoft Research Redmond, WA United States; 2 Idiographic Dynamics Lab Department of Psychology University of California Berkeley, CA United States; 3 MIT Media Lab Massachusetts Institute of Technology Cambridge, MA United States; 4 Siebel School of Computing and Data Science University of Illinois Urbana-Champaign Urbana, IL United States; 5 Paul G Allen School of Computer Science & Engineering University of Washington Seattle, WA United States; 6 Human-Centered Design and Engineering University of Washington Seattle, WA United States

**Keywords:** workplace stress, just-in-time, just-in-time adaptive intervention, JITAI, engagement, microintervention, stress reduction, psychotherapy

## Abstract

**Background:**

Integrating stress-reduction interventions into the workplace may improve the health and well-being of employees, and there is an opportunity to leverage ubiquitous everyday work technologies to understand dynamic work contexts and facilitate stress reduction wherever work happens. Sensing-powered just-in-time adaptive intervention (JITAI) systems have the potential to adapt and deliver tailored interventions, but such adaptation requires a comprehensive analysis of contextual and individual-level variables that may influence intervention outcomes and be leveraged to drive the system’s decision-making.

**Objective:**

This study aims to identify key tailoring variables that influence momentary engagement in digital stress reduction microinterventions to inform the design of similar JITAI systems.

**Methods:**

To inform the design of such dynamic adaptation, we analyzed data from the implementation and deployment of a system that incorporates passively sensed data across everyday work devices to send just-in-time stress reduction microinterventions in the workplace to 43 participants during a 4-week deployment. We evaluated 27 trait-based factors (ie, individual characteristics), state-based factors (ie, workplace contextual and behavioral signals and momentary stress), and intervention-related factors (ie, location and function) across 1585 system-initiated interventions. We built logistical regression models to identify the factors contributing to momentary engagement, the choice of interventions, the engagement given an intervention choice, the user rating of interventions engaged, and the stress reduction from the engagement.

**Results:**

We found that women (odds ratio [OR] 0.41, 95% CI 0.21-0.77; *P*=.03), those with higher neuroticism (OR 0.57, 95% CI 0.39-0.81; *P*=.01), those with higher cognitive reappraisal skills (OR 0.69, 95% CI 0.52-0.91; *P*=.04), and those that chose calm interventions (OR 0.43, 95% CI 0.23-0.78; *P*=.03) were significantly less likely to experience stress reduction, while those with higher agreeableness (OR 1.73, 95% CI 1.10-2.76; *P*=.06) and those that chose prompt-based (OR 6.65, 95% CI 1.53-36.45; *P*=.06) or video-based (OR 5.62, 95% CI 1.12-34.10; *P*=.12) interventions were substantially more likely to experience stress reduction. We also found that work-related contextual signals such as higher meeting counts (OR 0.62, 95% CI 0.49-0.78; *P*<.001) and higher engagement skewness (OR 0.64, 95% CI 0.51-0.79; *P*<.001) were associated with a lower likelihood of engagement, indicating that state-based contextual factors such as being in a meeting or the time of the day may matter more for engagement than efficacy. In addition, a just-in-time intervention that was explicitly rescheduled to a later time was more likely to be engaged with (OR 1.77, 95% CI 1.32-2.38; *P*<.001).

**Conclusions:**

JITAI systems have the potential to integrate timely support into the workplace. On the basis of our findings, we recommend that individual, contextual, and content-based factors be incorporated into the system for tailoring as well as for monitoring ineffective engagements across subgroups and contexts.

## Introduction

### Background

Work is a major source of stress in the United States, affecting more than half of Americans throughout most of the day [1]. Workplace stress leads to increased risk of mental and physical health disorders, decreased productivity and job satisfaction, and higher rates of accidents and employee costs [2-5]. Integrating stress reduction strategies directly into the workplace has proven to be effective and is widely recommended [6,7]. However, incorporating these individual-based techniques (eg, cognitive behavioral skills training, meditation, and exercise) [7,8] into the workday can be challenging due to work culture [9,10] or psychological barriers [9-11].

Information workers who frequently use computing technology face challenges, such as prolonged desk-bound and sedentary behaviors, that contribute to chronic physical and mental health issues [12,13]. High computer use without adequate breaks, high levels of multitasking, and constant connectivity demanded by information and communications technology has been found to be associated with increased stress and burnout [14-17]. Despite information and communications technology being associated with increased stress, workplace computing tools can also be leveraged to understand and reduce stress [18,19].

Passive sensing capabilities via ubiquitous devices have shown potential in health and well-being domains through monitoring and assessing individuals over time [20], and such data may be harnessed to provide precision mental health support. However, introducing new devices (eg, wearables) to an organization can be costly (eg, ≥US $100 per worker) and could be impractical for real-world, daily functional use with compliance and quality issues [21,22]. In contrast, everyday technologies commonly used at work (eg, webcam, keyboard, and software telemetry) can be harnessed for passing sensing and behavioral analysis, offering a more feasible approach to infer affect [23], physiology [24], attention [25], or stress [18,26].

Recently, technology-mediated support for mental health has generated interest for its ability to provide flexible and always-available access. However, these systems often provide generalized support that does not account for individual variabilities or contexts [27]. Despite an abundance of mental health apps [28,29], digital interventions that fit the specific workplace context are still highly sought after [27,30].

Digital mental health interventions (DMHIs), known as digital microinterventions, leverage technology to adapt existing evidence-based psychosocial interventions and leverage technology affordances to provide individual components of traditional psychotherapy focused on managing proximal symptoms (eg, relaxation for stress) in the hopes of achieving broad, distal objectives (eg, overcoming depression) [31]. Just-in-time adaptive interventions (JITAIs) have been introduced to deliver personalized, contextualized, and adaptable interventions using dynamic human behavior data captured through ubiquitous sensing technologies [27,32-34]. This concept has been explored in various health contexts, such as promoting physical activity, stress management, and weight management, with recent interest in applying them to positive coping skill use [35].

Despite their promises, JITAI systems are not yet pervasive with many applications still relying on ecological momentary assessments (EMAs [36]). Recent developments in algorithmic and machine learning approaches to dynamic adaptation have begun to show improvements in timing, receptivity, and engagement [37-40]. Operationalizing the adaptation of the system requires choosing appropriate tailoring variables and intervention options to drive the system’s decision-making regarding intervention timing and content. Prior research has examined factors associated with engagement in DMHIs [41,42], including personal-related (eg, demographics and personalities), content-related (eg, perceived fit and usefulness), and technology-related factors (eg, technical issues and privacy). However, most prior studies investigate study-long engagement, rather than examining “in-the-moment” engagement factors, which are crucial for improving the usability of interventions in real-world contexts [31], particularly because the integration of intervention use into life is a core facilitator for engagement [42].

A recent meta-analysis [36] has also shown that tailoring is an important aspect of JITAI design associated with greater efficacy. Despite JITAI systems’ potential to provide precision support [27], few studies demonstrate the value of just-in-time (JIT) support in improving user engagement [43,44]. Although the use of passively sensed data for contextual understanding and system adaptation is often recommended for the design of JITAI systems [27], many still rely mostly on EMAs, app use, or simple temporal features primarily from a single modality (ie, mobile devices) or without considering individual or intervention-related factors [36,44]. Passive sensing technologies offer numerous sets of contextual variables (eg, location, calendar, movement, and activity), presenting a challenge in designing sensing-capable JITAI systems: identifying a core set of tailoring variables among all possible variables that the system should consider for optimizing effective engagement [45]. Therefore, our primary goal and key contribution is to identify crucial tailoring variables that influence momentary engagement in digital stress-reduction microinterventions to inform the design of similar JITAI systems.

### Summary

In this study, we analyze engagement data from a 4-week longitudinal deployment of a workplace stress-reduction intervention system [19]. We leverage everyday workplace devices as unobtrusive and passive sensors to gain a glimpse into participants’ daily work activities (eg, emails, meetings, and computer activity). This system leverages the Cloud to integrate passively collected data and EMAs to deliver JIT nudges to engage in digital microinterventions across devices. Unlike laboratory experiments or controlled studies, our study allows observing users’ moment-by-moment interactions with the system in naturalistic work environments. We combine passively sensed work contexts, the system use, including which intervention participants chose and liked, individual demographics, personality traits, and coping styles to understand how the work context influences engagement patterns and to understand the appropriate conditions that lead to momentary intervention engagement and positive outcomes. We leverage such data from 43 participants to contextualize 1585 system-initiated interventions. From statistical modeling of the impact of individual, contextual, and intervention-related factors on engagement outcomes, we confirmed that individual factors (eg, age, gender, personality traits, and coping skills), as well as contextual and content-related factors (eg, availability and intervention modality), significantly influenced momentary intervention engagement, intervention choice, user ratings, and stress reduction outcomes. These findings suggest tailoring guidelines for JITAI systems whereby contextual and personalized factors can be used to find a positive balance between user preferences and maximal intervention efficacy.

In preparing this paper, we referred to the Guidelines and Checklist for the Reporting on Digital Health Implementations (iCHECK-DH) [46], which can be found in Multimedia Appendix 1.

## Methods

### Objectives

There are multiple considerations that influence JITAI systems’ decision points (ie, “a time at which an intervention decision is made” [32]), such as the right timing for a prompt, the right intervention for the moment, or the intervention likely to be engaged in. Therefore, this study aimed to identify factors that may contribute to improving participant engagement for JITAI systems. The study builds on a pilot implementation and deployment of a JIT stress-reduction microinterventions in a real-world workplace setting, targeting information workers who spend most of their working hours at processing information with computing devices [47]. The deployed system did not adapt the intervention content based on changing state or user context and, therefore, is not a full JITAI system. Instead, the study conducted a retrospective analysis of the deployment data to understand factors that influence the engagement and efficacy of workplace stress-reduction JITAI systems for future development.

### Participants

Information workers were recruited from a large technology organization via randomly distributed email advertisements. We recruited primarily US-based workers for the ease of system troubleshooting and to minimize any country-specific organizational factors. We enrolled participants on a first-come, first-served basis as long as we could satisfy system compatibility. To ensure system compatibility, interested participants completed a brief screener survey about their work setup (eg, primary device specification and operating system and web camera availability). Eligible participants, whose primary device specification met our sensing software requirements, were asked to install and run the study system on their primary desktop for 30 minutes. Only 43 participants that could run the sensing software on their desktop for ≥30 minutes were selected to participate in the study. The sample size of approximately 40 participants was determined for statistical power within a larger experimental study [19] that compared the JIT condition with a baseline condition for intervention effectiveness.

Our intake survey included several demographics measures such as age, gender, and role. Of the 43 participants, 29 (67%) identified as man. This distribution of gender closely aligns with the current industry demographics for large technology companies [48], and therefore, we consider this gender representation acceptable for our analysis. Of the 43 participants, 3 (7%) self-reported being aged 18 to 25 years old, 11 (25%) were 26 to 35 years old, 18 (42%) were 36 to 45 years old, 8 (19%) were 46 to 55 years old, 2 (5%) were 56 to 65 years old, and 1 (2%) was >66 years old. Moreover, 24 (56%) of 43 participants reported as being in engineering or development role, and 14 (33%) reported as being in sales or business strategy.

Other intake measures included the Depression, Anxiety, and Stress Scale-21 (DASS-21) [49]), the brief Big Five Personality Inventory-10 [50], the Emotional Regulation Questionnaire [51] designed to measure the tendency to regulate emotions through cognitive reappraisal and expressive suppression, and the 6-item brief resilience scale [52] that measures the ability to bounce back from stress. The average stress level of the stress subscale of DASS-21 reported by participants was 5 out of 21 (SD 3.8) which is within normal ranges. Our participants scored an average of 3.8 for agreeableness, 4.1 for conscientiousness, 2.6 for extraversion, 2.8 for neuroticism, and 3.5 for openness out of 5. Cognitive reappraisal was scored at 4.7 out of 7 (SD 1.1), expressive suppression was at 3.7 out of 7 (SD 1.3), and resilience was at 3.5 out of 5 (SD 0.9) on average.

### Study Implementation and Procedure

We deployed our system on the 43 participants who consented to the study, for a period of 4 weeks. During every workday of the 4-week study period, the system asked users to complete 5 EMAs per day during their reported work hours to capture their subjective stress ratings from the past 30 minutes (ie, “How would you rate your level of stress during the last 30 minutes?”). When the system determined that the user’s stress level may be high, the system sent JIT nudges via a chatbot, asking users to engage in a stress reduction microintervention. In the background, the system captured use data as well as passively sensed contextual data. Detailed description of the system architecture is provided in section 1 of Multimedia Appendix 2 [5,15,19,33,53-72].

### JIT Heuristics

The system determined higher-than-baseline stress levels based on our JIT heuristics informed by computed stress scores and self-reported stress levels. Stress scores were computed in real time per individual as an average of 5 components ranging between 0 and 1, each representing 5 components that previous work has identified as sources of stress: (1) the number of emails received [15,53], (2) the total number of meetings in a given day [54], (3) the percentage of time into the day [55], (4) the amount of facial expressions (via the Facial Action Coding System [56]) from corrugator (ie, brow furrowing) and lip depressor (ie, frowning) minus zygomatic major (ie, smiling) [55,57,58], and (5) heart rate [59,60]. Self-reported stress levels were obtained from EMAs.

In our JIT heuristics, first, we compute each user’s baselines as the average of the computed stress scores and self-reported stress levels based on the data from the first week of using the system. These individualized baselines (captured at week 1 of the 4-week study) are used as thresholds for delineating high stress from low stress during subsequent weeks (weeks 2 to 4). During the first week, we use the default baseline at the middle of the score range. Then, we send intervention nudges only if it is during the working hours that the users have stated at intake, if they have not explicitly scheduled an intervention at a later time that day, if they have not completed an intervention in the past hour, if there has not been an intervention nudge in the past 2 hours, and if there have not been ≥4 intervention nudges that day. Detailed description of our stress inference and heuristics is provided in section 1 of Multimedia Appendix 2.

### Microinterventions

Microinterventions [31] used in our study were translated from components of cognitive behavioral therapy and dialectical behavioral therapy, 2 empirically supported and widely used psychotherapy modalities [61,62]). These were under 5-minute interventions that were either a short video, a single-turn text prompt, or a brief therapeutic conversation with the chatbot. The microinterventions used in our study can be categorized by (1) the function served for users, (2) the modality in which the intervention was delivered, and (3) the intended location to perform the intervention.

Microinterventions are primarily categorized into 3 functional categories that align approximately with the amount of effort required. “Get my mind off work” interventions are low-effort interventions designed to help users take their mind away from work with positive activities that promote emotion regulation [63]. “Feel calm and present” interventions are medium-effort interventions that help users feel calm and present by drawing inspiration from the mindfulness practices. “Think through my stress” interventions are high-effort interventions that help users think through their stress and directly address and resolve stress-inducing components of their lives. For simplicity, we refer to these 3 intervention categories as “distract,” “calm,” and “address,” respectively, in the rest of the paper. Overall, there were 18 interventions per functional category. Section 2 of Multimedia Appendix 2 provides more details about the microinterventions, including all categories and examples for each category.

### User Engagement Flow

**Figure 1:**
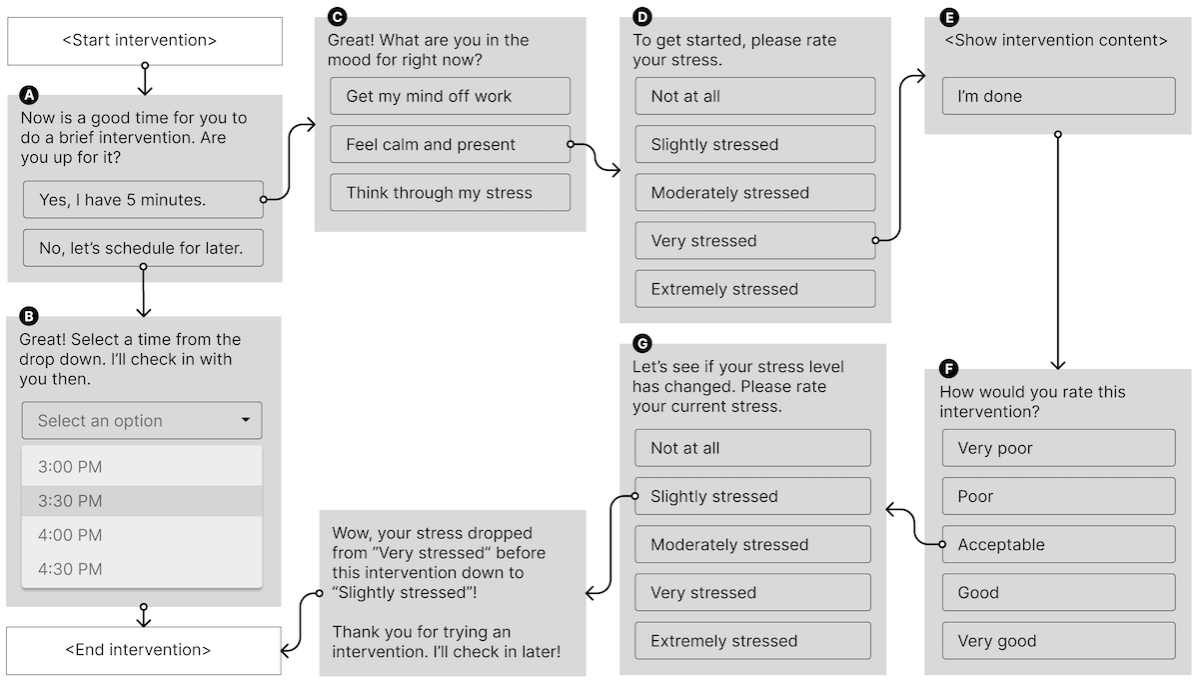
(A) System sends a nudge to users to perform an intervention. (B) Users can opt to postpone the intervention at a later time. (C) If users choose to do an intervention, they can select from 1 of 3 intervention categories. (D) Users first self-report their current stress level. (E) The system shows the intervention content for users to interact with. (F) User rates the intervention. (G) Users self-report their stress levels after the intervention.

Figure 1 illustrates a series of user engagement steps when a system nudge is sent. When the system sends a nudge to perform an intervention (Figure 1A), users can choose to delay it to a later time that day (Figure 1B). The system does not send another nudge until that time. Users may ignore the nudge, and the system expires the nudge after 30 minutes of inactivity. If the user decides to engage in an intervention, they can choose from 3 intervention categories (Figure 1C), and then the system randomly selects a new intervention within the category. Before beginning the intervention, the users are asked to subjectively rate their current stress level (Figure 1D). Then, the intervention content is shown to the user (Figure 1E). Once the intervention is done, the users are asked to rate the intervention (Figure 1F) and rate their stress level again (Figure 1G) before concluding the intervention flow. An example screenshot of the full user engagement flow can be found in section 3 of Multimedia Appendix 2.

### Other Study Tasks

Beyond JIT-based interactions with the system, participants could also access interventions on demand, where they could perform the intervention at that moment or schedule it to a later time that day. Participants were also asked to complete morning surveys to log their sleep quality and evening surveys to log their food and beverage intake. We additionally asked participants to rate their sleep quality the night before via a morning survey and log their food and drink consumptions throughout the day via an evening survey. They also completed weekly surveys including the DASS-21 and the brief resilience scales. The exit survey solicited feedback about the usability of the system and the perceived helpfulness and impact of the interventions.

### Poststudy Analysis

This section describes how we processed and analyzed the data collected from the above study to understand the factors contributing to improving participant engagement in a system-initiated intervention and the effectiveness of interventions at a given moment. Table 1 describes the full set of variables selected for our analysis and their descriptive statistics. Section 3 of Multimedia Appendix 2 includes additional detail, including descriptive statistics, inclusion or exclusion of data points, and correlational analyses conducted between variables.

**Table 1:** List of the per-participant, per-half hour, per-nudge, and per-intervention variables and their descriptive statistics.

Variable		Distribution
**Per-participant**		
	Age (y)	18-35 (n=14), 36-45 (n=18), >46 (n=11)
	Gender	Men (n=29), women (n=14)
	Cognitive reappraisal	Mean 4.69 (SD 1.09); range 2-7
	Expressive suppression	Mean 3.74 (SD 1.26); range 2-6
	Resilience	Mean 3.51 (SD 0.89); range 2-5
	Agreeableness	Mean 3.79 (SD 0.74); range 2-5
	Conscientiousness	Mean 4.12 (SD 0.83); range 2-5
	Extraversion	Mean 2.62 (SD 0.86); range 1-4
	Neuroticism	Mean 2.85 (SD 1.04); range 1-5
	Openness	Mean 3.48 (SD 0.79); range 2-5
	Engagement skewness	Mean –0.09 (SD 0.58); range –1.41 to 1.41
**Per-half hour**		
	Nudge probability	Mean 0.06 (SD 0.04); range 0.02-0.30
**Per-nudge**		
	Meeting counts	Mean 0.30 (SD 0.51); range 0-3
	No meeting minutes	Mean 5.43 (SD 11.79); range 0-50
	Self-event counts	Mean 0.12 (SD 0.37); range 0-3
	Email messages sent	Mean 0.39 (SD 0.88); range 0-8
	Email messages read	Mean 3.22 (SD 5.09); range 0-54
	Chat messages count	Mean 3.88 (SD 5.90); range 0-64
	Ad hoc call count	Mean 0.04 (SD 0.21); range 0-2
	Number of attention signals	Mean 1442.64 (SD 1316.68); range 0-5705
	Nudge source	JIT^a^ algorithm (n=1337), rescheduled (n=248)
	Engaged	True (n=563), False (n=1022)
**Per-intervention (chosen)**		
	Category	Address (n=112), calm (n=338), distract (n=200)
	Engaged	True (n=563), false (n=87)
	Modality	Video (n=113), prompt (n=384), conversation (n=24)
	Location	At desk (n=422), inside (n=84), outside (n=15)
**Per-intervention (completed)**		
	Stress reduction	Mean 0.29 (SD 0.53); range –1 to 3
	Rating	Mean 3.61 (SD 1.02); range 1-5
	Improved	True (n=150), false (n=371)
	Liked	True (n=289), false (n=232)
	Stress before	Mean 2.14 (SD 0.97); range 1-5
	Category	Address (n=77), calm (n=275), distract (n=72)
	Modality	Video (n=60), prompt (n=340), conversation (n=24)
	Location	At desk (n=325), inside (n=84), outside (n=15)

^a^JIT: just-in-time.

### Engagement Outcome Variables

From participants’ interactions with the system, we reconstructed each participant’s step-by-step interaction with the system as represented in Figure 1 to extract several outcome variables, such as whether a system-initiated intervention was completed and the effectiveness and rating of interventions once engaged. We note that the number of data points varies based on where in the engagement flow the participant exits.

#### Engagement Label

We labeled each system-initiated intervention as “engaged” in an intervention (ie, *engaged=*true) if the participant explicitly marked the intervention as done (ie, clicking on “I’m done” button in Figure 1E), regardless of whether they completed any subsequent prompts (ie, Figures 1F and G). Any ignored, incomplete, or timed-out nudges were considered not engaged. Each system-initiated intervention that was triggered at a postponed time (Figure 1B) was categorized as “rescheduled” (ie, *nudge source=*rescheduled). Although these rescheduled nudges looked identical to JIT nudges, we hypothesized that the participants would be more likely to engage in a system-initiated intervention if they postponed the intervention to a time that is more suitable for engagement.

#### Intervention Choice

The characteristics of interventions, such as location, modality, and effort level, are important to consider because certain interventions might not be feasible in certain situations (eg, the participant cannot go outside) [73,74]. If a participant chose an intervention category (Figure 1C), we marked that nudge with binary labels of *distract chosen*, *calm chosen*, or *address chosen*. Each of these interventions was further labeled with *modality* and *location* based on the specific intervention that the system chose within the category.

#### Intervention Effectiveness and Rating

We looked at 2 outcome metrics—momentary *stress reduction* and intervention *rating*. We further binarized these outcome metrics to determine if a certain intervention engagement *improved* stress (ie, self-reported stress rating was lowered after the intervention use) and if the participant *liked* the intervention (ie, rated as “good” or “very good”).

#### Tailoring Variables for Postdeployment Analysis

We used per-participant demographics and validated scale responses (coping skills and personality traits) to explain individual differences. We harnessed passively sensed data streams to explain the context surrounding the system initiation and intervention engagement. On the basis of the participants’ interactions with the system, we also account for the probability distribution of system nudges throughout the day and the participant’s likelihood of engaging in interventions in the morning or the afternoon.

### Participant Characteristics

We included demographic variables, such as age and gender, and personality traits that have been shown to impact engagement [42]. Because of the small sample sizes on either end of the age groups, we combined the lower 2 and upper 3 age groups to create more balanced age groups (ie, 18-35, 36-45, and >46 years). We included emotion regulation [51] and resilience [52] as measures of a person’s ability to cope with stressors. We also included the Big Five personality traits [75-77] because they are known to impact stress [78,79] and engagement in mental health treatment [42].

### Passively Sensed Context

To understand the context surrounding a system-initiated intervention, we leveraged 2 sources of passively sensed data: custom sensing software built by Microsoft Research and Viva Insights by Microsoft.

The custom sensing software ran on participants’ desktops and can capture activities that may not be associated with work, such as browsing the internet or using non–work related software. It captured data from the 5 components used to compute stress scores in real time, as described earlier. The sensing software also captured general user computer activity events such as mouse and keyboard interactions into a single metric, *number of attention signals*, which could be an important indicator for presence. We hypothesized that presence at the computer could lead to higher engagement in interventions as the nudges were designed to grab the attention of participants at work. The range of values for *number of attention signals* was fairly large (maximum=5705) compared with other variables, so we divided the values by 2000 to estimate a comparable coefficient and CIs during modeling (ie, to have odds ratios [ORs] within 2 decimal points). When interpreting the effect sizes, we corrected for this factor of 2000. We also hypothesized that the likelihood of engaging in a stress-reduction intervention during active participation in a meeting is low. Therefore, we included *no meeting minutes* to represent the total number of minutes without a scheduled meeting with others and *self-event count* as the total number of calendar events with only the participant as the attendee.

Viva Insights captures deidentified activity aggregates in 30-minute windows for Microsoft tools across all devices associated with an individual’s work account. From Viva Insights, we included *meeting counts*, *ad hoc call count*, and *email messages sent or read*. We excluded *chat messages count* from our analysis because the nudges were delivered through Microsoft Teams, and our data source cannot be used to discern if the messages were coming from the bot. Because Viva Insights data are limited to half-hourly windows, we associated the contextual metrics with each system-initiated intervention by taking the half-hour window that holds the nudge time stamp.

Both data sources had several overlaps or similar metrics, such as ones related to meetings or emails. Correlational analysis between 2 data sources is described in section 3 of Multimedia Appendix 2. It is important to note that contextual data at work can be noisy due to individual differences in the use of work tools for nonwork purposes (eg, personal use and subscriptions). We do not differentiate work versus nonwork data because it is challenging, for example, to isolate work-related emails as the only source of stress.

### System-Initiated Intervention Probability

Although each of the system nudges could be considered as an independent, repeated observation, the timing of the system-initiated interventions was sometimes dependent on when EMAs were administered. Because the system’s JIT heuristic runs every 5 minutes to check if a nudge needs to be sent based on the stress score and the EMA stress ratings, the most likely hours for receiving a system nudge is shortly after the EMA, leading to each participant receiving more nudges during certain hours of the day than others. To account for such variability in receiving system-initiated interventions, we incorporate the momentary nudge probability in our analysis. Because the range of nudge probabilities is small (mean 0.06, SD 0.04), we multiply the measure by 100 to represent it in percentages.

### Temporal Engagement Skewness

Prior research has found that different hours of the day were seen as good or not-so-good timing for stress interventions [33]. To examine if a certain participant has a temporal tendency to engage, we computed the Fisher-Pearson coefficient of skewness, or *engagement skewness*, on the hourly intervention engagements per participant. A positive *engagement skewness* means that the participants tend to engage at the beginning of their workday, and a negative *engagement skewness* means that the participants tend to engage toward the end of their workday. We used the skewness metric instead of simply looking at the engagement during the morning and the afternoon to account for individual differences in working hours. We incorporated this skewness per participant in our analysis.

### Analysis

Taking all the tailoring and outcome variables into account, our analysis focused on estimating the effects of contextual, individual, and intervention characteristics on binary outcome variables (eg, *engaged*, *distract chosen*, and *liked*). Thus, we built a logistic regression model predicting each outcome based on a combination of per-participant characteristics, per-half-hour nudge probability, per-nudge contextual metrics, or per-intervention metrics as fixed effects. The outputs of the logistical regression models are presented as ORs, representing a ratio of odds (eg, probability of engaging vs probability of not engaging) under 2 different conditions (eg, being a woman vs not being a woman). The data processing was conducted using Python packages (eg, *numpy*, *pandas*, *scipy*, and *seaborn*) and the models were tested using R libraries (eg, *lme4*, *car*, and *performance*).

We determined the significance of the fitted model against the null hypothesis model using the analysis of deviance. We conducted ANOVA to estimate the significance of fixed effects. Because the data are unbalanced (ie, unequal number of observations for each level of a factor), we obtain ANOVA type II sums of squares [80]. For categorical variables of ≥3 levels (eg, *age group* and *category*), we estimated pairwise differences using Tukey HSD (honestly significant difference) procedure. Multicollinearity in fixed effects was tested using the variance of inflation factor, and none of our models exhibited a multicollinearity issue. Results for ANOVA, multicollinearity, and Tukey analyses are presented in section 4 of Multimedia Appendix 2.

### Ethical Considerations

The study was reviewed by the Microsoft Research Institutional Review Board (OHRP IORG #0008066, IRB #IRB00009672) before the research activities and was formally approved. In addition to the ethics review, our study obtained approvals from Microsoft’s privacy, security, and legal review officers before conducting the study. All participants provided consent as part of the onboarding process and agreed for their deidentified data to be used for research purposes. The consent described the installation of our system, interaction with interventions, surveys, and joining the study data with cloud-based, device-independent telemetry data. All data, collected anonymously or otherwise, were collected and stored in a secured and access-controlled location. All data were joined and deidentified before analysis by the research team. For their participation and data, each participant was compensated with a US $400 Amazon gift card.

### Implementation Considerations

The implementation of the system used in the deployment study has dependencies that restrict interoperability and sustainability. The system requires that the participants use Microsoft platforms (eg, M365, Windows, Viva Insights, and Teams) and own a decent desktop that can perform vision-based data processing on device. Our current implementation focused on the understanding of individual, contextual, and intervention-related factors to drive the tailoring of JITAI systems and does not allow generalizability beyond the supported architecture. However, we believe that recent advancements in generative artificial intelligence technologies and the implementation of interoperability layers across different technology ecosystems can enable more accessible implementation of the system. Because this study was a pilot implementation, any budget planning, sustainability model, or interoperability for sustained deployment were not in scope for this paper.

## Results

### Intervention Engagement

To see which factors influenced engagement, we modeled *engaged* as a function of per-participant characteristics, per-half hour nudge probability, and per-nudge contextual metrics. The logistic regression model of *engaged* with all fixed effects was significantly different from the null hypothesis model (ie, engage ∼1; χ^2^_21_=115.5; *P*<.001). Table 2 outlines the ORs and CIs for each predictor. Predictors *engagement skewness*, *meeting count*, *number of attention signals*, and *nudge source* remained significant after applying the Benjamini-Hochberg correction for multiple comparisons. Reviewing the coefficients of our fixed effects, we found that being aged >46 years, being a woman, higher *cognitive reappraisal*, higher *number of attention signals*, and receiving a rescheduled nudge were associated with a higher likelihood of intervention engagement. In contrast, higher *engagement skewness* and *meeting count* were associated with to a lower likelihood of intervention engagement.

**Table 2:** Odds ratios (ORs) and CIs for each predictor of *engaged* for all system-initiated nudges^a^.

Predictors			Engaged, OR (95% CI)
Intercept			0.1^b^ (0.03-0.30)
**Age group (y), reference (18-35)**			
	36-45		1.17 (0.88-1.56)
	>46		1.47^c^ (1.07-2.02)
**Gender reference (man)**			
		Gender (woman)	1.37^c^ (1.05-1.79)
Cognitive reappraisal			1.14^c^ (1.01-1.29)
Expressive suppression			0.92 (0.83-1.02)
Resilience			1.05 (0.88-1.25)
Agreeableness			0.87 (0.72-1.05)
Conscientiousness			1.13 (0.96-1.33)
Extraversion			1.03 (0.89-1.21)
Neuroticism			0.97 (0.83-1.12)
Openness			1.15 (0.99-1.34)
Engagement skewness			0.64^b^ (0.51-0.79)
Nudge probability			1.02 (1.00-1.05)
Meeting counts			0.62^b^ (0.49-0.78)
No meeting minutes			1 (0.99-1.01)
Self-event counts			1.15 (0.85-1.54)
Email messages sent			1.05 (0.92-1.20)
Email messages read			1.01 (0.99-1.03)
Ad hoc call count			0.89 (0.52-1.49)
Number of attention signals			1.39^b^ (1.17-1.66)
**Trigger source reference (system)**			
	Trigger source (rescheduled)		1.77^b^ (1.32-2.38)

^a^Observations=1585; Tjur *R*^2^=0.07.

^b^Statistically significant (*P*<.05) after Benjamini-Hochberg correction.

^c^Statistically significant (*P*<.05) before any correction.

Next, we modeled each of the 3 choice outcome measures—*distract chosen*, *calm chosen*, and *address chosen*—as a function of per-participant characteristics, per-half hour nudge probability, and per-nudge contextual metrics. Table 3 outlines the ORs and CIs for all models.

**Table 3:** Odds ratios (ORs) and CIs for each predictor of *distract chosen, calm chosen,* and *address chosen* for all participant-chosen interventions.

Predictors			Distract chosen^a^, OR (95% CI)		Calm chosen^b^, OR (95% CI)		Address chosen^c^, OR (95% CI)
Intercept			0.11^d^ (0.01-0.73)		1.84 (0.32-10.62)		0.44 (0.04-4.52)
**Age group (y), reference (18-35)**							
	36-45	1.65^d^ (1.03-2.67)		0.62^d^ (0.40-0.96)		0.88 (0.46-1.65)	
	>46	0.71 (0.40-1.26)		0.95 (0.57-1.57)		1.6 (0.85-3.07)	
**Gender reference (man)**							
	Gender (woman)	0.62^d^ (0.39-0.98)		1.31 (0.87-1.96)		1.29 (0.73-2.26)	
Cognitive reappraisal			1.01 (0.82-1.25)		1.27^d^ (1.06-1.53)		0.67^e^ (0.53-0.84)
Expressive suppression			0.99 (0.83-1.19)		1.06 (0.90-1.25)		0.95 (0.75-1.18)
Resilience			0.93 (0.69-1.25)		0.87 (0.67-1.13)		1.42^d^ (1.00-2.02)
Agreeableness			0.75 (0.55-1.03)		1.02 (0.77-1.36)		1.46 (0.99-2.20)
Conscientiousness			1.30 (0.98-1.75)		0.92 (0.71-1.18)		0.74 (0.51-1.05)
Extraversion			1.18 (0.91-1.53)		1.00 (0.79-1.27)		0.79 (0.57-1.11)
Neuroticism			0.88 (0.68-1.14)		1.00 (0.79-1.26)		1.36 (0.99-1.89)
Openness			1.67^e^ (1.30-2.16)		0.71^d^ (0.57-0.88)		0.83 (0.60-1.13)
Engagement skewness			1.12 (0.74-1.68)		1.11 (0.78-1.59)		0.86 (0.53-1.38)
Nudge probability			1.00 (0.96-1.05)		1.01 (0.97-1.05)		0.97 (0.92-1.03)
Meeting counts			0.84 (0.55-1.25)		1.07 (0.76-1.52)		1.14 (0.71-1.77)
No meeting minutes			1.00 (0.99-1.02)		1.00 (0.99-1.01)		1.00 (0.98-1.02)
Self-event counts			1.08 (0.63-1.79)		0.83 (0.53-1.30)		1.19 (0.65-2.05)
Email messages sent			1.05 (0.84-1.30)		1.00 (0.82-1.22)		0.99 (0.74-1.30)
Email messages read			1.03 (0.99-1.07)		0.99 (0.96-1.03)		0.96 (0.90-1.01)
Ad hoc call count			0.88 (0.36-1.94)		0.93 (0.45-1.93)		1.26 (0.45-3.05)
Number of attention signals			0.65^e^ (0.48-0.87)		1.18 (0.92-1.53)		1.33 (0.94-1.87)
**Trigger source reference (system)**							
	Trigger source (rescheduled)	1.01 (0.61-1.63)		0.76 (0.49-1.16)		1.39 (0.81-2.35)	

^a^Observations=650; Tjur *R*^2^=0.115.

^b^Observations=650; Tjur *R*^2^=0.059.

^c^Observations=650; Tjur *R*^2^=0.089.

^d^Statistically significant (*P*<.05) before any correction.

^e^Statistically significant (*P*<.05) after Benjamini-Hochberg correction.

For choosing distract interventions, we found that higher *openness* was associated with a higher likelihood. In contrast, being a woman and higher *number of attention signals* were associated with a lower likelihood of choosing distract interventions. We found that higher *cognitive reappraisal* was associated with a higher likelihood and lower *openness* was associated with a lower likelihood of choosing calm interventions. We also found that higher *resilience* was associated with a higher likelihood and lower *cognitive reappraisal* was associated with a lower likelihood of choosing address interventions.

To understand the effect of intervention choice on engagement, we modeled *engaged* as a function of per-participant characteristics, per-half hour nudge probability, per-nudge contextual metrics, and per-intervention characteristics. The logistic regression model of *engaged* was significant (χ^2^_27_=69.6; *P*<.001). Table 4 outlines the ORs and CIs for each predictor. We found that a higher *resilience*, choosing a prompted-based intervention, and choosing a video-based intervention were associated with a higher likelihood of engagement. In contrast, choosing an intervention that could be performed inside was associated with a lower likelihood of engagement.

**Table 4:** Odds ratios (ORs), CI, and *P* values for each predictor of *engaged after chosen*, that is, engaged for all interventions after participants chose a category^a^.

Predictors		Engaged after chosen, OR (95% CI)
Intercept		0.05^b^ (0.00-0.964)
**Age group (y), reference (18-35)**		
	36-45	1.06 (0.54-2.05)
	>46	1.65 (0.71-4.02)
Gender (woman)		1.65 (0.88-3.16)
Cognitive reappraisal		1.11 (0.83-1.48)
Expressive suppression		1.25 (0.95-1.57)
Resilience		1.63^b^ (1.05-2.59)
Agreeableness		1.24 (0.77-2.00)
Conscientiousness		0.81 (0.55-1.17)
Extraversion		0.72 (0.48-1.08)
Neuroticism		1.39 (0.96-2.07)
Openness		1.24 (0.88-1.76)
Engagement skewness		1.01 (0.60-1.73)
Nudge probability		1.01 (0.95-1.07)
Meeting counts		0.65 (0.41-1.06)
No meeting minutes		1 (0.98-1.02)
Self-event counts		1.94 (0.86-1.67)
Email messages sent		1.16 (0.84-1.67)
Email messages read		0.97 (0.93-1.03)
Ad hoc call count		0.54 (0.23-1.33)
Number of attention signals		0.9 (0.61-1.33)
**Trigger source reference (system)**		
	Trigger source (rescheduled)	1.79 (0.85-4.10)
Engagement skewness		1.01 (0.60-1.73)
**Category reference (distract)**		
	Category (calm)	1.14 (0.60-2.14)
	Category (address)	0.55 (0.22-1.43)
**Modality reference (conversation)**		
	Modality (prompt)	3.53^b^ (1.35-9.60)
	Modality (video)	5.86^b^ (1.69-21.68)
**Location reference (at desk)**		
	Location (inside)	0.43^b^ (0.23-0.83)
	Location (outside)	0.34 (0.11-1.18)

^a^Observations=650; Tjur *R*^2^=0.12.

^b^Statistically significant (*P*<.05) before any correction.

### Intervention Effectiveness

Finally, we analyzed the factors associated with higher intervention rating and intervention effectiveness. The logistic regression model of *liked* as a function of per-participant characteristics, per-half hour nudge probability, per-nudge contextual metrics, and per-intervention metrics was significant (*χ^2^*_28_=92.4; *P*<.001). The ORs and CIs for each predictor can be seen in Table 5. We found that being a woman, higher *cognitive reappraisal*, higher *extraversion*, and higher *stress reduction* were associated with a higher likelihood of liking the intervention. In contrast, being aged 36 to 45 years, higher *expressive suppression* and higher *nudge probability* were associated with a lower likelihood of liking the intervention.

**Table 5:** Odds ratios (ORs), CIs, and *P* values for each predictor of *liked* for all interventions that participants engaged in^a^.

Predictors				Liked, OR (95% CI)
Intercept				5.95 (0.51-70.91)
**Age group (y), reference (18-35)**				
	36-45			0.51^b^ (0.30-0.86)
	>46			1.01 (0.55-1.83)
**Gender reference (man)**				
		Gender (woman)	2.51^c^ (1.51-4.20)	
Cognitive reappraisal				1.35^b^ (1.08-1.69)
Expressive suppression				0.82^b^ (0.68-0.99)
Resilience				1.10 (0.80-1.51)
Agreeableness				0.72 (0.50-1.02)
Conscientiousness				0.80 (0.58-1.09)
Extraversion				1.46^b^ (1.10-1.95)
Neuroticism				0.84 (0.64-1.11)
Openness				0.99 (0.75-1.30)
Engagement skewness				0.73 (0.47-1.12)
Nudge probability				0.94^b^ (0.90-0.99)
Meeting counts				0.93 (0.61-1.42)
No meeting minutes				1.00 (0.99-1.02)
Self-event counts				0.66 (0.40-1.10)
Email messages sent				1.01 (0.79-1.28)
Email messages read				1.00 (0.96-1.04)
Ad hoc call count				0.66 (0.24-1.69)
Number of attention signals				1.16 (0.84-1.59)
**Trigger source reference (system)**				
	Trigger source (rescheduled)			0.78 (0.47-1.30)
**Category reference (distract)**				
	Category (calm)			0.8 (0.49-1.29)
	Category (address)			0.94 (0.44-2.01)
	Stress reduction			2.36^c^ (1.60-3.54)
**Modality reference (conversation)**				
	Modality (prompt)			0.52 (0.17-1.53)
	Modality (video)			0.75 (0.22-2.47)
**Location reference (at desk)**				
	Location (inside)			0.69 (0.40-1.19)
	Location (outside)			2.56 (0.79-9.27)

^a^Observations=521; Tjur *R*^2^=0.166.

^b^Statistically significant (*P*<.05) before any correction.

^c^Statistically significant (*P*<.05) after Benjamini-Hochberg correction.

**Figure 2:**
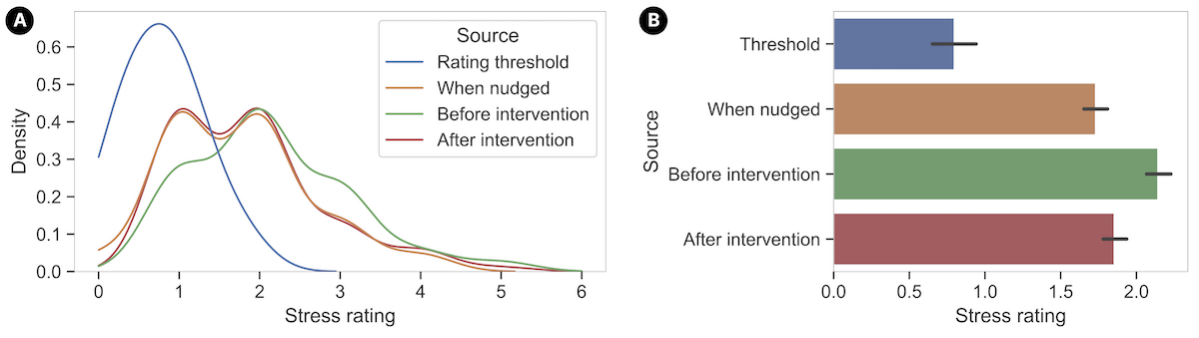
(A) Distribution of momentary stress ratings. A kernel density estimate plot of subjective stress ratings shows that the distribution of stress ratings when the system sent intervention nudges is higher than the individual thresholds. Stress ratings before intervention are distributed at a higher rating than stress ratings after intervention and the ratings when the nudges were sent. (B) Comparison of momentary stress ratings. Average momentary stress ratings at different points in time with 95% CIs.

When we examine the momentary stress ratings surrounding the system nudges and the intervention use (Figure 2A), we see that the distributions of EMA and preintervention and postintervention stress ratings sit higher than the individual thresholds used for sending the nudges. The average stress rating measured just before the intervention use (mean 2.14) is higher than the average stress rating used for system nudges (mean 1.73), indicating that the participants were more likely to engage in interventions when their stress ratings were higher than average (Figure 2B).

We then examined the factors associated with stress reduction. The logistic regression model of *improved* was significant (*χ^2^*_30_=186.7; *P*<.001), and the resulting ORs and CIs per predictor can be found in Table 6. We found that higher *agreeableness*, higher *nudge probability*, higher *stress before*, higher *rating*, and getting prompt-based or video interventions were associated with a higher likelihood of improvement on their stress rating. In contrast, being a woman, higher *cognitive reappraisal*, higher *neuroticism*, and choosing calm or address interventions were associated with a lower likelihood of improvement on their stress rating.

**Table 6:** Odds ratios (ORs), CIs, and *P* values for each predictor of *improved* for all interventions that participants engaged in^a^.

Predictors			Improved, OR (95% CI)
Intercept			0.00^b^ (0.00-0.00)
**Age group (y), reference (18-35)**			
	36-45		0.58 (0.30-1.13)
	>46		0.98 (0.49-1.98)
**Gender reference (man)**			
		Gender (woman)	0.41^b^ (0.21-0.77)
Cognitive reappraisal			0.69^b^ (0.52-0.91)
Expressive suppression			1.01 (0.79-1.28)
Resilience			0.84 (0.55-1.25)
Agreeableness			1.73^c^ (1.10-2.76)
Conscientiousness			1.27 (0.86-1.89)
Extraversion			0.89 (0.62-1.29)
Neuroticism			0.57^b^ (0.39-0.81)
Openness			1.09 (0.78-1.55)
Engagement skewness			1.18 (0.68-2.03)
Nudge probability			1.09^b^ (1.03-1.16)
Meeting counts			0.85 (0.48-1.42)
No meeting minutes			1.01 (0.99-1.03)
Self-event counts			1.29 (0.69-2.36)
Email messages sent			1.04 (0.78-1.39)
Email messages read			0.96 (0.90-1.01)
Ad hoc call count			0.94 (0.28-2.63)
Number of attention signals			1.4 (0.95-2.05)
**Trigger source reference (system)**			
	Trigger source (rescheduled)		1.08 (0.57-2.02)
**Category reference (distract)**			
	Category (calm)		0.43^b^ (0.23-0.78)
	Category (address)		0.40^c^ (0.16-0.97)
	Stress before		5.76^b^ (3.98-8.64)
	Rating		2.47^b^ (1.84-3.39)
**Modality reference (conversation)**			
	Modality (prompt)		6.65^c^ (1.53-36.45)
	Modality (video)		5.62^c^ (1.12-34.10)
**Location reference (at desk)**			
	Location (inside)		1.17 (0.60-2.25)
	Location (outside)		0.23 (0.03-1.10)

^a^Observations=521; Tjur *R*^2^=0.338.

^b^Statistically significant (*P*<.05) after Benjamini-Hochberg correction.

^c^Statistically significant (*P*<.05) before any correction.

## Discussion

### Principal Findings

In this paper, we presented a comprehensive and systematic study that simultaneously encompassed trait-based factors (ie, individual characteristics), state-based factors (ie, workplace contextual and behavioral signals and momentary stress), and intervention-related factors (ie, location and function) to identify what drives JIT intervention engagement and efficacy. This study particularly focuses on momentary outcomes to inform the design of *dynamic tailoring*, which is a key component of JITAI systems. In this study, we leveraged surveys, EMA, and passively-sensed data from the deployment of the JIT stress-reduction intervention system to identify significant factors that influence the momentary engagement, the choice of interventions, the engagement given an intervention choice, the user rating of interventions engaged, and the stress reduction from the engagement.

We found that stress ratings immediately after the JIT interventions were significantly lower than those reported in the moments immediately before the interventions (Figure 2B). While keeping in mind the study sample, we found that women, those with higher *neuroticism*, those with higher *cognitive reappraisal* skills, and those that chose *calm* and *address* interventions were significantly less likely to experience stress reduction, while those with higher *agreeableness* and those that chose prompt-based or video-based interventions were significantly more likely to experience stress reduction. Surprisingly, contextual signals, such as meeting or email load, did not lead to a significant increase or decrease in stress ratings, which may indicate that trait-based or intervention-related factors matter more for efficacy or that the contextual signals may need finer granularity.

In contrast, we found that contextual signals such as lower *meeting counts* and lower *engagement skewness* were associated with a higher likelihood of engagement, indicating that state-based contextual factors such as being in a meeting or time of the day may matter more for engagement than efficacy. In addition, a JIT intervention that was explicitly rescheduled to a later time was more likely to be engaged. This implies that intervention engagement can be improved by giving some level of control to the users over complete automation.

With respect to the choice of interventions and liking the interventions, factors that significantly influenced the choice were primarily trait-based. Higher *openness* was associated with a higher likelihood of choosing *distract* but a lower likelihood of choosing *calm*, while higher *cognitive reappraisal* was associated with a higher likelihood of choosing *calm* but lower likelihood of choosing *address*. Higher *resilience* was associated with a higher likelihood of choosing *address* as well as the subsequent engagement after the choice. Interventions were liked more by women, those with higher *cognitive reappraisal*, and those with higher *extraversion*, and when the interventions led to stress reduction. One state-based factor that stood out is the *number of attention signals*, which negatively impacted the likelihood of choosing *distract* interventions. Higher *number of attention signals* indicates higher desk-bound work activity (eg, keyboard and mouse) where getting the mind off work (ie, how *distract* interventions were communicated) during intense work activities may not be feasible or desired.

In summarizing our findings, we first categorize these factors into (1) nonmodifiable individual factors, (2) modifiable individual factors, (3) contextual factors, and (4) content factors.

The distinction between modifiable and nonmodifiable individual factors is important for intervention design. Once modifiable factors are identified, strategies can be deployed to directly influence those factors if those strategies can lead to a greater impact on the engagement or the efficacy of the interventions. Nonmodifiable factors are also important to determine which subset of populations can benefit from additional targeted support through organizational or policy-level changes [81]. In our analysis, nonmodifiable individual factors include gender and age, and modifiable individual factors, although debatable [82], include emotion regulation skills and personality traits. Our findings revealed that these individual factors not only influence study-long engagement [42] but also influence momentary engagement and can be useful for tailoring JITAI systems. Although our findings for individual factors corroborate with prior studies, it is important to highlight that our study evaluates instance-level engagement metrics through play-by-play analysis of app-use behaviors rather than study-long engagement metrics. Contextual factors such as workload (eg, meeting counts and email counts) and availability (eg, presence or activity at the computer) are helpful in the JITAI system’s decision-making process for when to interrupt the user. Content factors include intervention-related information such as the amount of effort required, the modality of intervention delivery, and the ideal location for intervention engagement, and these factors can inform JITAI systems in determining which intervention to present to the user given the understanding of the current context.

In this section, we summarize and discuss the findings organized by these 4 categories of factors with recommendations for design and future research.

### Nonmodifiable Individual Factors

In our analysis, we found several significant effects of gender and age on our outcome measures. Participants who self-reported as being a woman had more than twice the likelihood of liking interventions than being a man, despite having less likelihood of improving from engaging in interventions. Being a woman also had 38% less likelihood of choosing *distract* interventions than being a man, suggesting that there may be an unobserved motivational factor. For example, although not statistically significant, women reported higher momentary stress on average compared with men (2.15 for women vs 1.84 for men; *t*_20.756_=–1.545; *P*=.14), which may contribute to choosing more *calm* or *address* interventions to reduce their stress. Although the general findings from DMHI studies that women are more likely to engage in digital interventions than men are corroborated by our analysis [42], the fact that women improve less despite engaging more, liking interventions more, and choosing more *address* (ie, high reward) interventions is a concern for systems design that only take engagement metrics into account.

Participants aged >46 years were 47% more likely to engage in interventions while being aged 36 to 45 years increased the likelihood of choosing *distract* interventions by 65% and decreased the likelihood of liking interventions by 49% compared with those aged 18 to 35 years. Although the effects of different age groups on engagement have mixed results across prior studies, the higher engagement rate for participants aged >46 years in the study could be explained by a higher rate of interest in digital interventions for older populations [83]. Participants in the 36 to 45 years age group choosing *distract* interventions and not liking them highlight an opportunity for finding different types of interventions that they might enjoy.

Tailoring JITAI systems to individuals has been suggested to improve engagement and efficacy of interventions in prior research [36]. Our findings suggest that certain age or gender groups may benefit more than others from our system. The lack of engagement or the lack of improvement despite engagement for some groups highlights opportunities for targeting research and design efforts to further understand unobserved barriers to engagement and effective responses. Although it is generally recommended that intervention content be tailored based on individuals, we also recommend that the efficacy and user rating of interventions be closely monitored to ensure that certain groups enjoy the same level of benefits as other groups. In addition, new intervention content could be codesigned with groups that may not be benefiting as much and added to the system on a regular basis to equalize outcomes across subgroups.

### Modifiable Individual Factors

Our findings corroborate prior research that personality trait is a strong factor in the engagement of DMHIs [42]. A prior study found that openness to experience is associated with better adherence and lower odds of attrition [84]. In our analysis, we did not find a significant effect on engagement, but we found that *openness* significantly increases the likelihood of choosing *distract* but decreases the likelihood of choosing *calm* interventions. One possible explanation may be the variety in the intervention content, which people with high openness would prefer [85]. *Distract* interventions tend to offer more variety in content with videos of travel destinations and humor as well as opportunities to explore social connections, whereas *calm* interventions were mostly introspective activities such as breathing or focused observations.

Although the effects were only moderately significant (*P*<.06), we found that an increase by one point in *neuroticism* and *agreeableness* scales increases the likelihood of choosing *address* interventions, which were designed to help users directly address resolve the stress-inducing components of their lives. For participants with high scores in *neuroticism* and *agreeableness* scales, it is possible that interventions that help them directly address their stress were more appealing than others that were designed to distract from stress and refocus on the present. Prior study has also found neuroticism and agreeableness to associate with a stronger interest in the use of stress management apps [86]. We also found that one point increase in *agreeableness* scale was associated with an increased likelihood of improvement by 73%, whereas the same point increase in *neuroticism* scale was associated with a lower likelihood of improvement by 43%, despite both having higher tendency to choose *address* interventions. Because agreeableness is known to be positively associated with the therapeutic alliance in mental health treatments [87], it is possible that the prosocial and cooperative nature of those with high agreeableness [88] allowed them to fully engage in the *address* interventions that were more action-oriented, leading to a greater improvement. In contrast, neuroticism has been known to negatively correlate with adherence to mental health recommendations [89] and to a wide variety of mental health treatment outcomes [87].

Prior work has associated extraversion with lower interest in using web-based mental health services over face-to-face interactions with a provider [83]. Although our interventions were delivered solely through technology, we found that one point increase in *extraversion* scale increases the likelihood of liking the intervention by 46%. We found no significant association between the intervention content (category, modality, or location) and liking them to explain this behavior. Future research should explore how extraversion facilitates momentary engagement in interventions.

Corroborating with prior study-long findings that associate personalities with DMHI engagement, our study found that personality traits also influence momentary engagement and efficacy of interventions. We recommend that JITAI systems carefully monitor potentially unhelpful use behaviors by incorporating personality traits in the system adaptation algorithm because they may impact the choice of interventions that may lead to negative downstream effects on outcomes. For example, it may be beneficial to offer a variety of more effortful interventions for people who report higher scores in openness. For people who report higher scores in neuroticism, the system could suggest less effortful interventions.

Across the board, emotion regulation styles had significant effects. One point increase in the *cognitive reappraisal* scale was associated with a 14% increase in the likelihood of engaging, a 27% increase in the likelihood of choosing *calm* interventions, a 35% increase in the likelihood of liking the intervention, a 33% decrease in the likelihood of choosing *address* interventions, and a 31% decrease in the likelihood of improvement after engagement. It is important to note the differences in the range of point scales. An increase of 14% for a 7-point scale is equivalent to an increase of 20% for a 5-point scale. In contrast, one point increase in the *expressive suppression* scale was associated with an 18% decrease in the likelihood of liking the intervention. We also found that one point increase in the *resilience* scale increases the likelihood of choosing *address* interventions by 42% and increases the likelihood of subsequently engaging in interventions by 63%.

Prior research has explored the role of emotion regulation in stress coping. For example, emotion regulation skills help assess stressful situations and determine the appropriate emotional response [90] or act as buffers against the negative effects of stress [91]. Emotion regulation has also been theorized as a moderator for increased resilience after encountering a stressful situation [92]. Although our analysis cannot claim the causal direction between coping skills and engagement, our findings suggest that emotion regulation and resilience may play a role in not only the stress-coping process but also in choosing different interventions or deciding to engage in an intervention. While most research has argued for increasing coping skills as an outcome measure or a treatment target [93], our findings suggest promising new research directions in understanding how coping skills could impact our decisions to engage in therapeutic interventions in the moment.

The role of emotion regulation strategies in altering our decisions and choices in various contexts has been empirically studied in highly controlled laboratory settings [94]. Prior research has also studied personality traits [82,95] and coping skills [93] as “states” that exhibit intrapersonal variations, and such states can be modified through therapeutic strategies (eg, cognitive behavioral therapy). Although our study has assumed personality traits, emotion regulation skills, and resilience to be stable over the course of the study, it remains to be seen how shifting the perspectives of these characteristics to be more dynamic would inform the JITAI systems. Because of the potential mediating role of coping skills in perceived stress [96] and the role of perceived stress in outcome improvement (ie, one point increase in stress rating before the intervention leads to being >5 times more likely to improve in our findings), coping skills should be measured periodically and incorporated into the decision-making process of JITAI systems. Therefore, we recommend further research to explore how taking a dynamic approach to personality and coping skills would inform the design of JITAI systems.

### Contextual Factors

We found that the more the participant tends to engage at the end of the day, the more likely they would engage in interventions and that the system-initiated interventions that were rescheduled to a later time increased the likelihood of engagement. These findings may suggest a tendency to defer interventions to later in the day. Prior work that applied the self-determination theory [97] to JITAI systems suggested that perceived competence and self-regulation abilities may deplete throughout the day, potentially leading to unhealthy choices (eg, unhealthy food and alcohol) toward the end of the day [34]. This has a serious consequence for those who tend to procrastinate or postpone healthy behaviors (eg, exercise, and stress intervention) toward the end of the day. In contrast, allowing people to defer an intervention to a specific time may increase self-efficacy and perception of control, which might lead to an increased chance of behavior change [98]. In fact, Howe et al [19] reported that many participants liked the ability to perform the interventions when they wanted. We also found that the increase in nudge probability decreases the likelihood of liking the intervention but increases the likelihood of improvement, revealing that a JIT intervention might be “a bitter pill to swallow” but a useful pill, nonetheless. However, a relentless reminder could lead to distraction [99] and eventual system abandonment [44].

Our findings show encouragement that intelligent timing based on contextual information could improve the engagement and effectiveness of interventions, but perhaps at the cost of a lowered sense of user agency and control, negative perceptions toward the interventions, or leading to unhealthy choices toward the end of the day. It also highlights that relying solely on what people like may not be helpful for stress reduction and that there needs to be an additional investigation into how momentary factors could influence user ratings. Therefore, future JITAI systems should carefully balance user ratings (eg, likes) with intervention efficacy (eg, stress reduction and engagement) and help users discover what works best for them. Considering that higher user ratings may not always reflect the effectiveness of the interventions, such systems should aim to simultaneously explore the rating and the improvement in determining the timing of interventions.

In evaluating the work context, as expected, we found that a nudge sent at a time when the user is less likely to be in a meeting but more active at the desk improves engagement. Contrary to our hypothesis, we found no significant associations with *no meeting minutes* or *self-event counts*. It is possible that there is high variability in the level of focus and attention needed during times carved out for self. For example, our data sources cannot discern if the times carved out for self were work-related (eg, focus time for reading and writing) or non–work related (eg, running errands, child pick up, and exercise). This study’s data sources cannot achieve automatic detection of activities beyond basic work activities, such as meetings, emails, chats, calls, or computer activities.

Although tailoring to the activity context is the defining promise of JITAI systems, automatically detecting the activities performed within a time window is not an easy task. In addition, how much tailoring and the level of system intelligence are really necessary to maximize engagement and outcomes is unknown, especially given the cost and risks associated with the invasion of privacy in passive sensing. Further research is necessary to understand the cost and benefit of accurate activity detection and intelligent timing on the engagement and effectiveness of JITAI systems.

### Content Factors

In our analysis, intervention categories, modalities, and locations showed pronounced effects on engagement and improvement, suggesting the importance of the intervention content in the design of JITAI systems. We found that having an intervention that could be done at the desk more than doubled the likelihood of engagement compared with an intervention that could be done indoors, but not at the desk. It is possible that leaving the desk at the moment of the nudge was not appropriate given the situation or there was an unobserved motivational barrier. In these scenarios, suggesting a different activity, rescheduling the activity, or waiting until the next appropriate transition time might have been beneficial. To improve engagement, intervention designers could provide additional desk-based stress reduction techniques to minimize the burden of leaving the desk.

In contrast, we found that having a prompt- or video-based intervention increased the likelihood of engagement by more than 3-fold and improvement by more than 5-fold, compared with a conversation-based intervention. Although prompt- and video-based interventions were typically less effortful than conversation-based interventions that require many turn-taking interactions with the bot, conversation-based interventions were designed to address the sources of the stress with the hope of creating a longer-lasting impact. It is possible that the conversations were not usable for participants to fully engage in the content. This finding suggests that quick, effortless interventions could be useful at the moment, but complex, turn-taking interventions need more thoughtful redesign.

Our findings revealed that the effects of intervention content types were significant and large. To investigate the impact of choices on improving outcomes and user ratings, future digital intervention systems that provide a catalog of interventions would benefit from characterizing each intervention on multiple dimensions, such as the level of effort, location, modality, etc. Such characterization would help understand the interplay among personality traits, contextual cues, and intervention types that users are likely to choose and benefit from. Therefore, we recommend that JITAI systems provide a variety of interventions to fine-tune its recommendations based on contexts but also to identify interventions that may need redesign.

### Limitations

Our system design and analysis setup have several limitations. The retrospective analysis of the inferred stress scores indicates that there is room for improvement. Our stress scores were computed from a generalized algorithm, but recent studies have shown that stress can be idiosyncratic and, therefore, needs to be modeled at an individual level [18]. Our user engagement flow cannot differentiate the dismissal of the nudge due to bad timing or low stress or both. Our analysis setup does not allow for determining the causal relationship between the individual, contextual, and content factors with engagement, stress reduction, and intervention ratings. Even though the deployment study collected long-term stress measures via the DASS-21 scale, our analysis was limited to momentary stress ratings because microinterventions are more appropriate for proximal outcomes over distal outcomes [31]. Microrandomized trials are a promising research direction for JITAI systems to quantify the impact of tailored interventions on both short- and long-term outcomes [100] with careful considerations for the appropriate sample size [101]. Our data were also limited by a small sample population that exhibited low-stress levels, focusing on US-based information workers from a large technology organization, skewed toward engineers and those that identified as man. Prior work has suggested that, when evaluating engagement (or attrition), the severity of symptoms should also be considered [41]. Therefore, future research should evaluate the system through microrandomized trials with a sample population exhibiting high severity of stress symptoms while also expanding into other information worker roles from different sectors.

The deployment of the system used in the study is limited by the system requirements. Our system assumes that a single organization may offer a standardized set of software tools to workers for several reasons (eg, security, privacy, productivity, compliance, and scalability) and does not support interoperability across different software tools used by workers within an organization or across organizations. Beyond this challenge, it is important to note that the data required to describe an individual worker’s stress necessarily crosses work–non-work boundaries, and such boundaries are increasingly blurred by technologies [102] and remote work [103]. Our contextual data captured at work were noisy and were aggregated without peeking into the content (eg, of email or documents) to preserve privacy; in addition, it is challenging to separate blurred contexts. Such blurred boundaries raise concerns surrounding boundary preferences, data ownership, values and incentives, well-being definitions, and power dynamics that may undermine the successful deployment of personal stress support systems [104]. Perhaps more important than the interoperability of a system or clean data is a human-centered implementation process that allows for flexibility and adaptation of the system based on multistakeholder perspectives and use over time. This paper piloted one aspect of such adaptation. However, much work is needed in realizing the full potential of JITAI systems that take sociotechnical considerations into account and where users can participate in drawing the boundary and providing the right level and fidelity of data that meets their needs.

### Conclusions

JITAI systems have the potential to integrate timely support into the workplace. To identify key factors that should be incorporated into the system’s adaptable decision-making process, we analyzed data from a 4-week deployment of a JIT workplace stress reduction microintervention system. On the basis of our findings, we recommend that individual, contextual, and content-based factors be incorporated into the system for tailoring as well as for monitoring unhelpful use behaviors across subgroups and contexts. Future work should explore careful balancing of individual preferences, intervention efficacy, and system accuracy to help users discover what works best for them and to continuously improve system recommendations.

## Appendix

Multimedia Appendix 1 Completed Checklist of Guidelines and Checklist for the Reporting on Digital Health Implementations (iCHECK-DH).

Multimedia Appendix 2 (1) Detailed system architecture and stress inference methods, (2) intervention design and example interaction flows, (3) detailed description of how interaction and context data were extracted, and (4) additional results from ANOVA type II and multicollinearity analyses conducted for each logistic regression model.

## Abbreviations

DASS-21: Depression, Anxiety, and Stress Scale-21
DMHI: digital mental health intervention
EMA: ecological momentary assessment
iCHECK-DH: Guidelines and Checklist for the Reporting on Digital Health Implementations
JIT: just-in-time
JITAI: just-in-time adaptive intervention
OR: odds ratio

## Data Availability

The data sets generated during or analyzed during this study are not publicly available due to privacy and legal restrictions but are available from the corresponding author on reasonable request with a clear justification and a license agreement. The request will be reviewed and approved case by case by Microsoft Research Release and Compliance team, at which point a license agreement will be drafted and shared.
