# Supplementary material for: Toward Tailoring Just-in-Time Adaptive Intervention Systems for Workplace Stress Reduction: Exploratory Analysis of Intervention Implementation
Source: JMIR Ment Health. 2024 Sep 12;11:e48974. doi: 10.2196/48974 (PMC11427862; doi:10.2196/48974)
Supplement: Multimedia Appendix 1 [file mental_v11i1e48974_app1.pdf]

Multimedia Appendix 1

Completed checklist of iCHECK-DH guidelines for manuscript "Towards Tailoring Just-in-time Adaptive Intervention Systems for Workplace Stress Reduction: Analysis of Intervention Implementation"

SECTION		ITEM	DESCRIPTION OF CHANGE
TITLE	1	Title (M[1])	Titled "Towards Tailoring Just-in-time Adaptive Intervention Systems for Workplace Stress Reduction: Exploratory Analysis of Intervention Implementation"
ABSTRACT	2	Abstract (M)	Abstract includes implementation strategy (JITAI systems), intervention (stress-reduction micro-interventions) and key objectives (identification of tailoring variables for JITAI systems that improve engagement and effectiveness) in the recommended order.
INTRODUCTION	3	Context (M)	Introduction includes the context of workplace stress management and the opportunity for technology-enhanced adaptation of interventions (Developing or Adapting Solution).
	4	Problem statement (M)	Introduction includes description of the problem of workplace stress and challenges of incorporating interventions into the workday and the opportunity for leveraging everyday technologies for intelligent adaptation of interventions.
	5	Similar Interventions (M)	Introduction includes description of several concepts for novel interventions, such as JITAI, micro-interventions, and passive-sensing.
METHODS	6	Aims and Objectives (M)	Method includes a section titled "Objectives" that describe study aims and objectives.
	7	Blueprint summary (M)	Method includes a section titled "Study implementation and procedure" that provides a high-level blueprint for the intervention. Multimedia Appendix 1 includes a more detailed description of the system, intervention, and tailoring variables used for the implementation.
	8	Technical Design (M)	Method includes a section titled "Study implementation and procedure" that provides a high-level blueprint for the intervention. "Multimedia Appendix 1" includes a more detailed description of the system, intervention, and tailoring variables used for the implementation.
	9	Target (M)	Method includes a "Participants" section that describes the target population as well as considerations around exclusion in the "Implementation considerations" section.
	10	Data (M)	Method includes an "Ethical consideration" section that describes the data governance and consent process, with brief discussion around data ownership and privacy challenges surrounding the system in "Discussion".
	11	Interoperability (M)	Method includes a section titled "Study implementation and procedure" that provides a high-level blueprint for the system. "Multimedia Appendix 1" includes a more detailed description of the system used for the implementation. Method includes a section titled "Implementation considerations" to discuss the limitations of the study.
	12	Participating entities (M)	As the study is a pilot for a novel intervention, participating entities still remain general as a technology organisation. Method includes a section titled "Implementation considerations" to discuss the limitations of the study.
	13	Budget Planning (M)	As the study is a pilot for a novel intervention, budget planning is not conducted. Method includes a section titled "Implementation considerations" to discuss the limitations of the study.

[1] M: Mandatory item

	14	Sustainability<br>(M)	As the study is a pilot for a novel intervention, sustainability effort is not conducted. Method includes a section titled “Implementation considerations” to discuss the limitations of the study.
**RESULTS**	15	Coverage<br>(M)	Method includes a “Participants” section that describes the coverage to be a US-based large technology organisation. As the study is a pilot for a novel intervention, broad coverage consideration is out of scope.
	16	Outcomes<br>(M)	Results section includes a summary of modelling outputs, where tailoring variables are used to predict several outcomes, such as stress reduction, engagement, or choice of interventions.
	17	Lessons learned<br>(M)	As the study is primarily a post-hoc data analysis, implementation lessons are enumerated in the “Discussion” section as recommendations.
	18	Unintended consequences<br>(NM[2])	Discussion includes a “Limitations” section that discusses sociotechnical implications of deploying the intervention.
**DISCUSSION**	19	Conclusion<br>(M)	Discussion interprets the data and draws recommendations for future JITAI system design and development.
**GENERAL**	20	General<br>(NM)	Method includes the “Ethical considerations” section that details ethical approval regarding the study.

[2] NM : Non-mandatory item
