# Supplementary material for: Toward Tailoring Just-in-Time Adaptive Intervention Systems for Workplace Stress Reduction: Exploratory Analysis of Intervention Implementation
Source: JMIR Ment Health. 2024 Sep 12;11:e48974. doi: 10.2196/48974 (PMC11427862; doi:10.2196/48974)
Supplement: Multimedia Appendix 2 [file mental_v11i1e48974_app2.pdf]

Multimedia Appendix 2

Towards Tailoring Just-in-time Adaptive Intervention Systems
for Workplace Stress Reduction:
Exploratory Analysis of Intervention Implementation

1 System Architecture

The primary design objective for the intervention system is to maximize intervention engagement in the workplace context by reducing the burden of data collection and by bringing interventions to where the workers are. To achieve these goals, we leverage passively and unobtrusively sensed data about work contexts from data sources that span across modalities and devices that are common and readily available at work. We also surface interventions whenever and wherever work happens to be. The system is optimized for capturing salient signals about work (Section 1.2) and inferring workers' stress levels (Section 1.5) where workers are more likely to be in front of their computers. On the other hand, it offers flexibility through capturing telemetry data from the usage of cloud-based and device-independent tools (Section 1.1) and through communicating via the chatbot that can be accessed from computers or mobile devices (Section 1.3). These data sources and experiences are coordinated by a cloud-based orchestrator (Section 1.4) to compute a stress score (Section 1.5) and to determine the timing for JIT interventions (Section 1.6).

1.1 Device-independent telemetry

Cloud-based workplace productivity tools such as Outlook, Teams, Word, Excel, PowerPoint, etc. are used to conduct work on desktops, laptops, or mobile devices. Such telemetry data can be collected and aggregated by the service providers across devices to provide rich user experiences (e.g., a list of recently accessed files, recommended files for an upcoming meeting) or for personal consumption. For instance, Microsoft Graph APIs allow users to access their own data [1]. Examples of computed metrics include the number of emails sent or received, the number of meetings, usage of chats and calls, the number of cloud-based documents accessed, etc. In our deployed system, we obtained this data via Microsoft's Viva Insights [2] and leveraged the telemetry data only retrospectively after the study was concluded. In future systems, the relevant telemetry information may be sent to the cloud-based orchestrator (Section 1.4).

1.2 Unobtrusive desk-bound sensors

Common workplace tools such as a webcam, keyboard, or mouse are used to conduct work in desk-bound situations. Signals from these tools and applications are collected and aggregated by a custom Windows desktop sensing software installed on the user's desktops or laptops. The video feed from the webcam can be used to infer facial expressions and physiological states (e.g., heart rate, breathing rate) using a non-contact measurement technique [3]. From peripheral devices, keyboard or mouse-related signals such as keystroke speed or mouse movements can be computed.

Application usage signals such as foreground or background applications or the number of window switching can also be computed. The same sensing software can tap into the user's account to access cloud-based device-independent telemetry (Section 1.1). In our deployed system, we used Outlook to compute email and calendar-related metrics on the device, and all computed metrics were sent to the cloud-based orchestrator using Azure Service Bus (Section 1.4).

1.3 Chatbot

A chatbot interacts with the users for data collection (e.g., EMAs) and for intervention content delivery. In our deployed system, we use Teams as a host for our chatbot such that messages can be accessed anywhere that work happens, including desktops, laptops, web browsers, and mobile devices. The chatbot interfaces with the orchestrator (Section 1.4) to maintain its conversation states and to receive instructions for engaging with users. The chatbot was implemented using Microsoft's Bot Framework [4]. Most prompts and responses are designed using Adaptive Cards [5], but some content (e.g., video-based interventions, weekly surveys) were implemented as Teams task modules (i.e., embedded web controls) to provide a seamlessly integrated experience all within Teams.

1.4 Cloud-based orchestrator

Signals, metrics, and data across devices as well as interactions with the chatbot can be sent to a central orchestrator that stores and manages the data as well as run computations and logic to drive user experiences that the chatbot affords. In our deployed system, the orchestrator was an Azure-hosted system made up of several components. A service bus receives data from multiple sources and saves the data in cloud storage that hosts received and computed data. A scheduler runs every 5 minutes to coordinate timings for EMAs based on the study configuration. It also runs the JIT heuristics (Section 1.6) over the information accessible from cloud storage to determine if now is an opportune moment to surface interventions. Finally, a chatbot runtime holds the conversational states and logic for the chatbot and provides secure web APIs for interfacing with the storage.

1.5 Stress inference

Given contextual data and individual characteristics, the stress inference algorithm can compute a stress score – a likely level of stress that a user may currently experience – where a high score could be an opportune time for interventions. Prior work has shown [6] that computer usage, workload, intervention history, and heart rate variability are helpful for predicting intervention timing for stress reduction. Ideally, this computation is done within the cloud-based orchestrator. However, in our deployed system, the computation was done by the custom sensing software installed on the user's desktop or laptop and sent to the orchestrator as a prototype implementation. Instead of a machine-learned stress inference, we use a simple and explainable estimate using an additive function. The stress score was computed every 30 seconds.

The stress score is an average of 5 components ranging between 0 and 1, each representing five components that previous work has identified as sources of stress: (1) the number of email received [7, 8], (2) the total number of meetings in a given day [9], (3) the percentage of time into the day [10], (4) the amount of facial expressions (via the Facial Action Coding System [11]) from

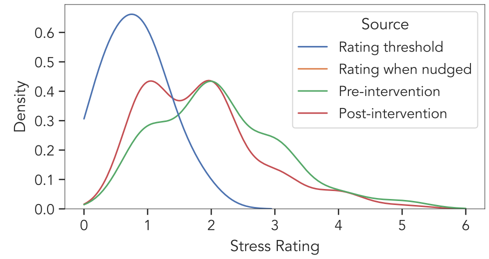

(a) Distribution of momentary stress ratings

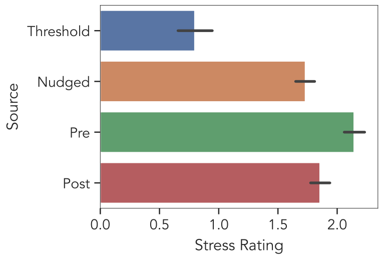

(b) Comparison of momentary stress ratings

Figure S1: (a) A kernel density estimate plot of subjective stress ratings shows that the distribution of stress ratings when the system sent intervention nudges is higher than the individual thresholds. The pre-intervention stress ratings are distributed at a higher rating than post-intervention stress ratings and the ratings when the nudges were sent. (b) Average momentary stress ratings at different points in time with 95% confidence intervals.

corrugator (i.e., brow furrowing) and lip depressor (i.e., frowning) minus zygomatic major (i.e. smiling) [12, 10, 13], and (5) heart rate [14, 15].

When we examine the momentary stress ratings surrounding the system nudges and the intervention use (Figure S1a), we see that the distributions of EMA, pre-intervention, and post-intervention stress ratings sit higher than the individual thresholds used for sending the nudges. The average stress rating measured just before the intervention use ($\bar{x}$=2.14) is higher than the average stress rating used for system nudges ($\bar{x}$=1.73), indicating that the participants were more likely to engage in interventions when their stress ratings were higher than average (Figure S1b).

A retrospective analysis of the correlation between our stress scores and participants' self-reported ratings on their momentary stress levels via EMAs (1=Not at all stressed; 3=Moderately stressed; 5=Extremely stressed) showed a significant positive correlation between the two (N=1981, Pearson r=0.14, p$\ll$0.001). The correlations between stress scores and self-reporting ratings during interventions were lower, with Pearson r=0.05 (p=0.19; not statistically significant) for pre-intervention stress ratings and Pearson r=0.09 (p=0.02) for post-intervention stress ratings.

For inferred stress scores, even though the distribution of scores when the nudges were sent was higher than the individual thresholds (Figure S2a), the average stress scores just before the intervention use ($\bar{x}$=0.46) were lower than the average scores when the nudges were sent ($\bar{x}$=0.51, Figure S2b) unlike what we observed with the subjective stress ratings. Although the inferred stress scores are not perfect, our heuristics leverage both sources of stress measures (i.e., EMA and stress score) to make the decision to send the intervention nudge. In addition, we augment potential system errors by giving users flexibility and agency to access interventions on demand and to reschedule nudges to a later time, which we describe next.

1.6 JIT heuristics

All available information, such as contextual data, individual characteristics, and subjective stress rating, can be used to determine if the system should nudge the user to perform an intervention. In our deployed system, we rely on both stress scores and self-reported stress levels to inform the JIT heuristics to improve the reliability of the system and to maximize the potential efficacy of the stress intervention [16]. For example, the stress score may not always be available because the participant

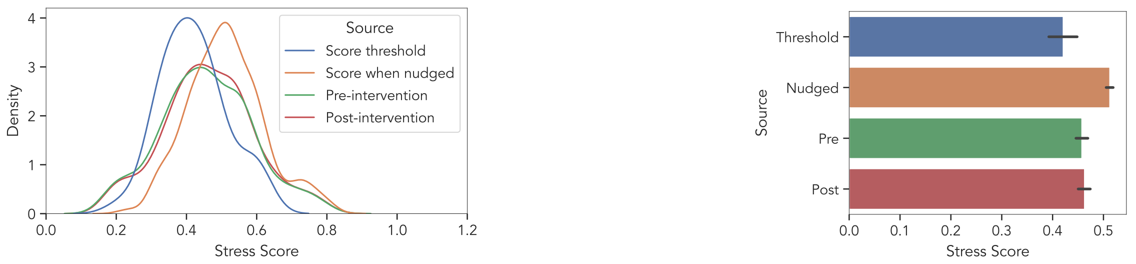

(a) Distribution of inferred stress scores

(b) Comparison of inferred stress scores

Figure S2: (a) A kernel density estimate plot of inferred stress scores shows that the distribution of stress scores when the system sent intervention nudges is higher than the individual thresholds as well as the scores before and after the intervention use. (b) Average inferred stress scores at different points in time with 95% confidence intervals.

may have disabled the sensing software temporarily or moved away from their desk. Or, the EMAs may not be available because the participant may have ignored the prompt or might be too busy to engage.

The heuristics are designed to incorporate individual differences in baseline stress levels. More importantly, the heuristics ensure that the system does not aggressively prompt for engagement to avoid burdening the users [17] and that the user preferences for interventions (e.g., work hours, prescribed timing) are respected. First, we compute each user's baselines as the average of the computed stress scores and self-reported stress levels based on the data from the first week of using the system. These individualized baselines (captured at week one of the four-week study) are used as thresholds for delineating high-stress from low-stress during subsequent weeks (weeks two to four). During the first week, we use the default baseline at the middle of the score range. Then, we send intervention nudges only if it is during the working hours that the participants have stated at intake, if they have not explicitly scheduled an intervention at a later time that day, if they have not completed an intervention in the past hour, if there has not been an intervention nudge in the past two hours, and if there have not been four or more intervention nudges that day.

Because our orchestrator runs the JIT heuristics every 5 minutes, there were approximately 185,000 logged instances across all participants where higher than threshold stress levels were detected. Only 0.79% of these instances proceeded to actual nudges sent to the participants due to the throttling logic we described above. In our deployment, we found 67.4% of the nudges sent by the system were driven by the computed stress score crossing the individual stress score threshold, and the rest were driven by the subjective stress ratings. The distribution of the stress scores and subjective stress ratings when the system sends the nudges against the individual thresholds can be seen in Figure S1 and Figure S2.

2 Intervention Design

2.1 Micro-interventions

Micro-interventions used in our study were translated from components of Cognitive Behavioral Therapy (CBT) and Dialectical Behavioral Therapy (DBT), two empirically supported and widely

used psychotherapy modalities [18, 19]). These were under five-minute interventions that were either a short video, a single-turn text prompt, or a brief therapeutic conversation with the chatbot. The micro-interventions used in our study can be categorized by (a) the function served for users, (b) the modality in which the intervention was delivered, and (c) the intended location to perform the intervention. Table S1 lists all categories of micro-interventions and an example for each category. Overall, there were 18 interventions per category.

Table S1: Intervention categories and examples.

Category Type	Category	Example
Function	*Distract*	Immerse yourself underwater in the Great Australian Bight.
	Calm	Breathe in and out. Stay focused on the sensation of the air coming into your air passages and lungs, holding it, and then letting it out. Count each breath until you reach 10. If you lose count, start over until you reach 10.
	Address	Weigh pros and cons to improve your motivation at work.
Modality	*Video*	Research shows that laughing can improve your physical and mental health. Check out this video. I dare you: Try not to laugh!
	Prompt	Identify something stressful that happened in your life in the past 24 hours. Now, imagine that this stressful thing happened to a friend. What would you say to them to make them feel better? Type it here.
	Conversation	Chat with your wellness bot to shift your thoughts about a stressful situation.
Location	*At desk*	Choose a single, short task you need to accomplish. Pause all notifications on your devices. Set a 15 minute timer. While the timer is going, work on the task you identified and nothing else. Resist any urges to leave your work station or check your phone or email – all of that can wait for 15 minutes. When the timer goes off, answer these questions: (1) Did you complete the task? and (2) How do you feel?
	Inside	Identify the nearest sink. Walk over to it, turn on the cold water as cold as it can be and let it run over your hands for 30 seconds. Then, turn the water as hot as possible for 30 seconds. Notice how your hands feel under each condition. Change the water back and forth 3-4 times before returning to your work station. Note, if you are living in a location with water shortages, you can do this activity by holding and then setting down a single ice cube. When you're finished, come back and tell me what you noticed.
	Outside	Stand up, walk outside. Stand outside for a few minutes. Reach up to the sky, and then down to your toes. Walk back inside. What did you notice outside?

Functional categories Micro-interventions are primarily categorized into three functional categories which align approximately with the amount of effort required. 'Get my mind off work' interventions are low-effort interventions designed to help users take their mind away from work with positive activities that promote emotion regulation [20]. 'Feel calm and present' interventions are medium-effort interventions that help users feel calm and present by drawing inspiration from the mindfulness practices. 'Think through my stress' interventions are high-effort interventions that help users think through their stress and directly address and problem-solve stress-inducing

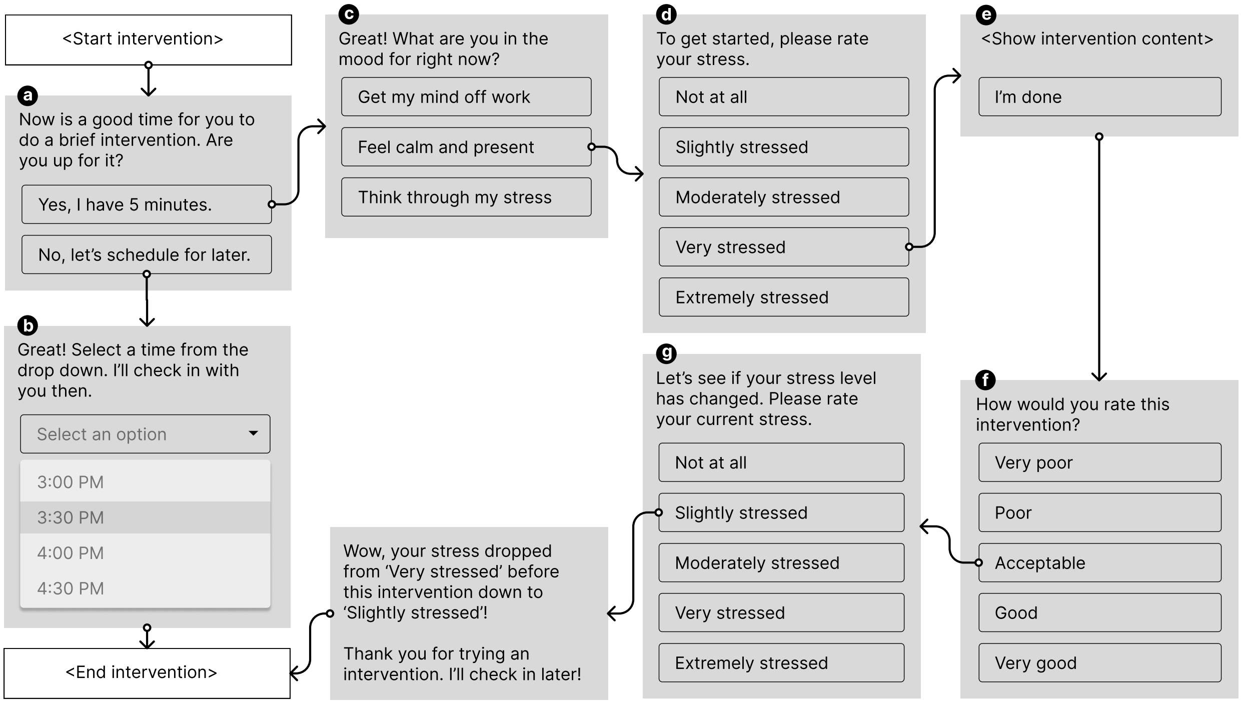

Figure S3: (a) System sends a nudge to users to perform an intervention. (b) Users can opt to postpone the intervention at a later time. (c) If users choose to do an intervention, they can select from one of three intervention categories. (d) Users first self-report their current stress level. (e) The system shows the intervention content for users to interact with. (f) User rates the intervention. (g) Users self-report their stress levels after the intervention.

components of their lives. For simplicity, we refer to these three intervention categories as 'Distract', 'Calm', and 'Address', respectively, in the rest of the paper.

Modality categories The system provides micro-interventions in three modalities. Video-based interventions open an embedded video player to show calming or funny videos. Prompt-based interventions provide a set of instructions, followed by a prompt that the user must respond to. Conversation-based interventions require turn-taking with the chatbot to answer a series of reflective questions.

Location categories While all micro-interventions can be performed anywhere the chatbot is accessible, some interventions explicitly encourage being outside or accessing materials or locations away from the primary work station. Outside interventions require being physically outside (e.g., going for a walk) and observing nature. Inside interventions often require the users to get up from the desk and to access different parts of the room (e.g., light exercise on the floor) or to change the environment (e.g., lighting). At desk interventions do not require leaving the current work context and can be done with tools that are typically utilized for work (e.g., watching videos, doodling).

2.2 User engagement flow

Although intelligent prompt-based systems (e.g., ESM, JIT) should always strive to find the right time to engage with the users and take into account other tasks that compete for their attention (e.g., phone calls or driving [17]), it is impractical to assume that any system is perfect, and a fully automated but imperfect system may erode user trust and lead to poor user experience. Therefore, the design of such systems should allow users to take control when the system is wrong or even when the system is right [21].

In our deployment, while the system was primarily responsible for determining when to send an automated nudge, there was a series of steps involved in interacting with the users to provide them with a level of flexibility and agency in proceeding with the nudge or in determining the appropriate function that the intervention should serve (i.e., the categories in Section 2.1). Therefore, both the system and the user made a series of choices during the engagement flow.

When the system sends a nudge to perform an intervention (Figure S3a), users can choose to delay it to a later time that day (Figure S3b) if the timing is not right or if the user thinks of a better time to engage in interventions. Once the user reschedules the intervention to a later time, the system fully respects that choice by not sending another nudge until that time. Users may ignore the nudge intentionally or unintentionally, and the system expires the nudge after 30 minutes of inactivity.

If the user decides to engage in an intervention, they can choose from three intervention categories that best suit their current need (Figure S3c). Once the user chooses a category, the system randomly selects an intervention. We intentionally prioritized suggesting interventions that are least used to promote the exploration of different content across various modalities and types. With 18 interventions per category across 20 days of the study, this approach minimizes the likelihood of repeated interventions.

Before beginning the intervention, the users are asked to subjectively rate their current stress level (Figure S3d). Regardless of whether the user is currently in a high- or low-stress state, the system respects their decision to engage in an intervention because prior research shows that practicing skills is an important reminder for using them in the moment of need [22]. Then, the intervention content is shown to the user (Figure S3e). Once the intervention is done, the users are asked to rate the intervention (Figure S3f) and rate their stress level again (Figure S3g) before concluding the intervention flow. An example screenshot of the full user engagement flow can be found in Figure S4.

3 Interaction and Context Extraction

To understand the context in which participants engaged in system-initiated interventions and the effectiveness of interventions at a given moment, we extracted several data. From participants' interactions with the system, we determined whether a system-initiated intervention was completed and the effectiveness and rating of interventions once engaged. We harnessed passively sensed data streams to explain the context surrounding the system initiation and intervention engagement.

3.1 Engagement label

We labeled each system-initiated intervention as "engaged" in an intervention (i.e., *Engaged* = TRUE) if the participant explicitly marked the intervention as done (i.e., clicking on "I'm done" button in

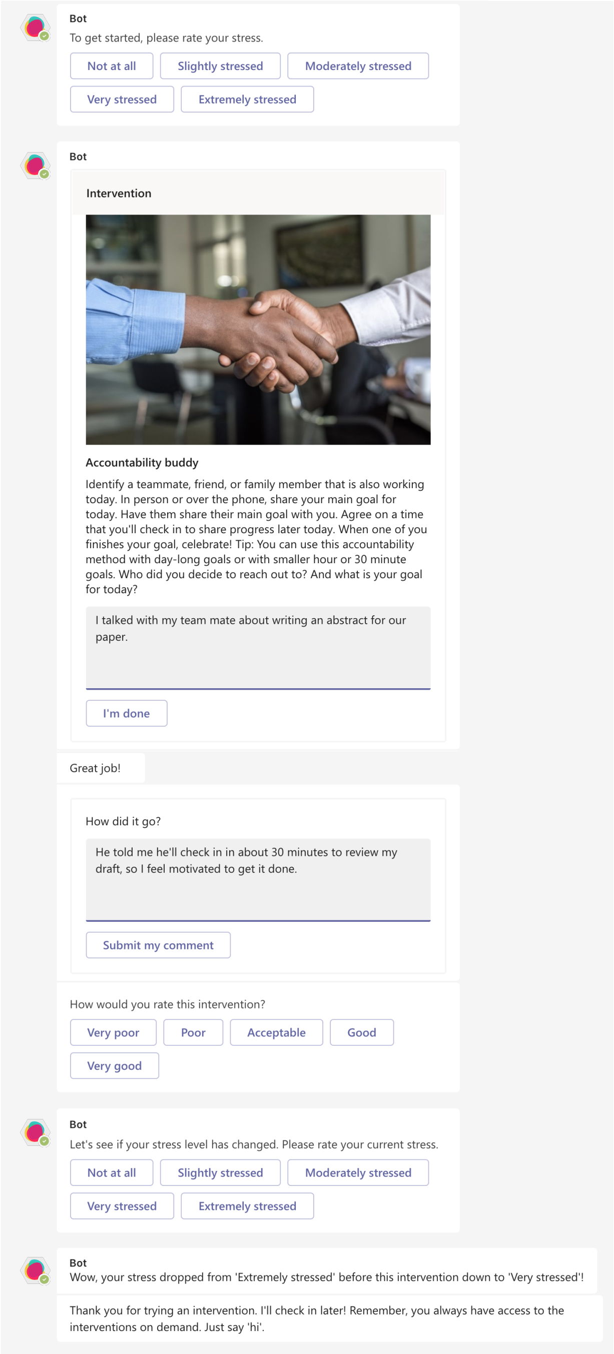

(a) Desktop experience

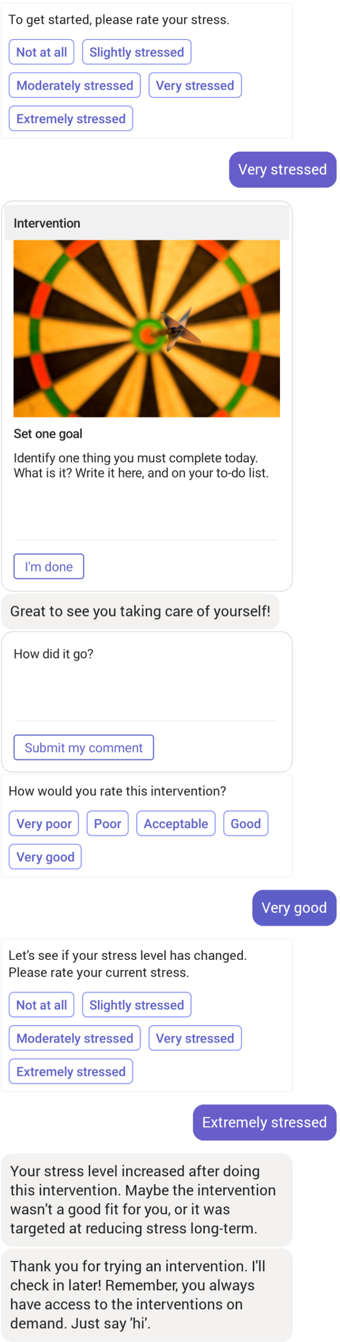

(b) Mobile device experience

Figure S4: Screenshots of the intervention user engagement flow with the chatbot within Microsoft Teams across the (a) desktop experience and the (b) mobile experience.

Figure S3e), regardless of whether they completed any subsequent prompts (i.e., Figure S3f-g). Any ignored, incomplete, or timed-out nudges were considered not engaged.

As shown in Figure S3b, participants can opt to postpone the intervention to a later time. Each system-initiated intervention that was triggered at this later requested time was categorized

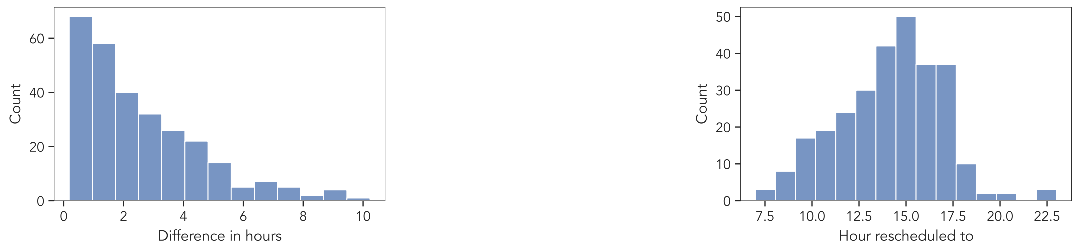

(a) The number of hours between the initial nudge and the rescheduled time

(b) Time of the day when the intervention is rescheduled to

Figure S5: (a) A histogram of the number of hours between the initial nudge and when the intervention is rescheduled to. (b) A histogram of the time of the day when the intervention is rescheduled to.

as "rescheduled" (i.e., *Nudge Source* = Rescheduled). Although these rescheduled nudges looked identical to JIT nudges, we hypothesized that the participants would be more likely to engage in a system-initiated intervention if they remembered having postponed the intervention to a time that is more suitable for engagement. On average, these rescheduled interventions were rescheduled to 2.6 hours after the nudge (σ=2.0, mode=2.0). 78.9% of the rescheduled interventions were scheduled at noon or after. Figure S5 illustrates the distribution of the reschedule delay and the time of day that the intervention was rescheduled to.

We found that 91.3% of nudges were responded to within 30 minutes of the nudge. Each nudge is canceled by the system if there had not been a participant response in 30 minutes and if there were outstanding EMA requests. Once participants began conversing with the bot, 96.8% of the engaged interventions were completed within 30 minutes of the first interaction with the bot. To better capture the context surrounding the intervention engagement, we associated each engagement label with a timestamp from the first interaction with the bot. For cases where the participant ignored the nudge, we associated each engagement label with a timestamp of the initial nudge.

Because our goal is to understand the influence of workplace contextual factors, we limited our analysis to weekdays (Monday-Friday). We further excluded days that participants explicitly stated as being out of the office for the entire day to remove noise introduced by atypical weekdays. As a result, there was a total of 1,585 system-initiated interventions with available context data, and 35.5% (563) of those nudges led to a completed intervention engagement.

3.2 Intervention choice

If a participant chose an intervention category (Figure S3c), we marked that nudge with binary labels of *Distract Chosen*, *Calm Chosen*, or *Address Chosen*. Each of these interventions was further labeled with *Modality* and *Location* based on the specific intervention that the system chose within the category. *Modality* is one of three options – video, prompt, or conversation. Of all the interventions presented to participants, there were 113 video-based, 384 prompt-based, and 24 conversation-based interventions. *Location* refers to whether the intervention could be performed at the desk, anywhere inside, or outside. There were 422 interventions that were done at the desk, 84 inside, and 15 outside. There were 650 total instances with category labels, and 563 (86.6%) of those were "engaged" interventions.

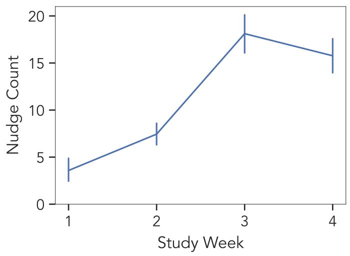

(a) Number of system-initiated interventions

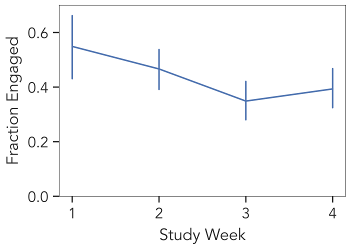

(b) Fraction of intervention nudges engaged

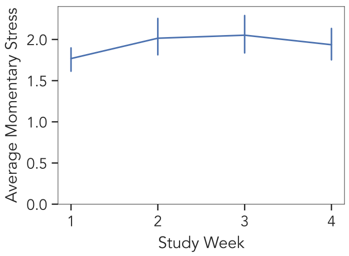

(c) Average momentary stress rating

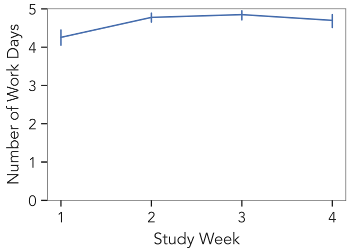

(d) Number of work days

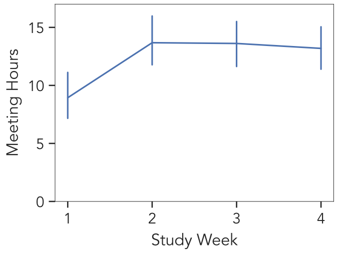

(e) Number of meeting hours

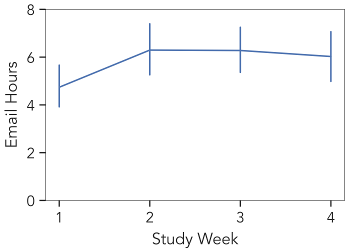

(f) Number of email hours

Figure S6: Weekly trend of (a) the number of system-initiated interventions, (b) the number of intervention engagements, (c) the average momentary stress ratings (1=Not at all; 2=Slightly stressed; 3=Moderately stressed), (d) the number of work days and workload, (e) the number of hours spent in meetings, and (f) the number of hours spent in attending to emails. Error bars indicate 95% confidence intervals from bootstrapping (n=1000).

3.3 Intervention effectiveness and rating

Of 563 system-initiated interventions that were engaged, 521 (92.5%) had both pre- and post-intervention stress ratings and intervention ratings. 275 of these interventions engaged were Calm interventions, 169 were Distract interventions, and 77 were Address interventions.

We looked at two outcome metrics – momentary *Stress Reduction* and intervention *Rating*. The average stress rating before engaging in the intervention was 2.14 (σ=0.97) on a 5-point scale (1=Not at all stressed; 5=Extremely stressed). On average, each intervention engagement led to a momentary stress reduction of 0.29 (σ=0.89) which was found to be statistically significant according to the paired t-test ($t(520)$=-12.20, p$\ll$0.001). Each intervention engagement was rated on average at 3.61 (σ=1.02; 1=Very poor; 5=Very good).

We further binarized these outcome metrics to determine if a certain intervention engagement *Improved* stress (i.e., self-reported stress rating was lowered after the intervention use) and if the participant *Liked* the intervention (i.e., rated as 'Good' or 'Very good'). In our data, *Stress Reduction* and *Rating* were positively correlated (Pearson r=0.214, p$\ll$0.001). As such, the binarized outcomes, *Improved* and *Liked*, were also significantly associated (χ^2(1)=18.839, p$\ll$0.001).

3.4 System-initiated intervention probability

Throughout the 4 week study, the average number of interventions in which participants engaged increased from 1.61 (σ=1.66) per participant at week 1 to 5.84 (σ=3.98) at week 4. This increase could be plausibly attributed to the increased number of nudges sent to the participants as the study progressed (Figure S6a). Week 1 of the study happened to be the week following a major US holiday (4th of July) when many employees were more likely to recharge after vacations [23]. The average number of work days per participant during week 1 was 4.33 (σ=1.02), compared to 4.90 during week 3 (σ=0.37, Figure S6d). Our data revealed that the general workload was lower immediately following the holidays. In fact, the meeting volume significantly increased throughout the study (β=1.269, F(1)=7.479, p=0.007, Figure S6e). The average total hours of meetings per week increased from 8.94 (σ=6.41) at week 1 to 13.19 (σ=6.38) at week 4 (t(82.0)=-3.048, p=0.003, Figure S6e). The average total hours of email per week increased from 4.74 (σ=2.91) at week 1 to 6.03 (σ=3.52) at week 4 (t(79.2)=-1.827, p=0.071, Figure S6f).

The system determined the threshold for triggering interventions based on the individual baselines captured at week 1. Both the increased workload and self-reported stress ratings are likely to have caused the average number of nudges per participant to increase after the first week. Although the number of intervention engagements increased with the increased number of nudges, the fraction of nudges that led to engagement decreased over time with 0.549 (σ=0.385) at week 1 to 0.393 (σ=0.242) at week 4 (Figure S6b), indicating that there may be an upper limit to the number of nudges that will lead to engagements. In fact, the weekly number of nudges and the fraction of engagements were negatively correlated (Pearson r=-0.319, p=1.786e-06). Therefore, when modeling the factors that contribute to engagement in system-initiated interventions, we also consider the general probability of receiving a nudge in the following way.

On average, participants received 48.37 (σ=15.29) system nudges and performed 18.2 (σ=9.48) interventions during the 4-week study. Although each of these nudges could be considered as an independent, repeated observation, system-initiated interventions were not uniformly distributed throughout the day due to particulars of the system design. The system nudges partially depended on self-reported stress levels which were captured through EMAs. These EMAs were spread out evenly between the fixed start and end work hours of each participant, typically 8 AM and 5 PM. Because the system's JIT algorithm runs every 5 minutes to check if a nudge needs to be sent based on the stress score and the EMA stress ratings, the most likely hours for receiving a system nudge is shortly after the EMA, leading to each participant receiving more nudges during certain hours of the day than others (Figure S7a). To account for such variability in receiving system-initiated interventions, we incorporate the momentary nudge probability in our analysis. Because the range of nudge probabilities is small ($\bar{x}$=0.06, σ=0.04), we multiply the measure by 100 to represent it in percentages.

3.5 Temporal engagement skewness

Prior research has found that different hours of the day were seen as good or not-so-good timing for stress interventions [6]. Howe et al. [24] reported that participants typically scheduled interventions at the beginning or the end of the day, indicating that there may be individual preferences for when to engage in interventions. To examine if a certain participant has a temporal tendency to engage, we computed the Fisher-Pearson coefficient of skewness, or *Engagement Skewness*, on the

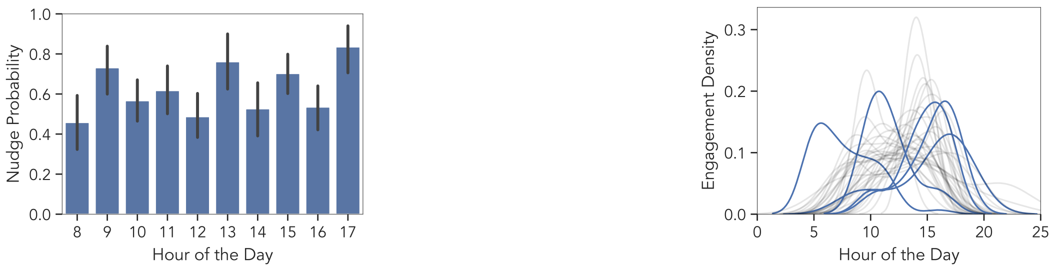

(a) Probability of receiving a nudge

(b) Intervention engagement density

Figure S7: (a) Probability of receiving a system-initiated intervention during typical work hours (8 AM - 5 PM). Error bars indicate 95% confidence intervals from bootstrapping (n=1000). (b) Kernel density estimate for engaging in a system-initiated intervention. Blue lines indicate participants for whom the Fisher-Pearson coefficient of skewness is significant ($p < 0.05$).

hourly intervention engagements per participant. A positive *Engagement Skewness* means that the participants tend to engage at the beginning of their workday, and a negative *Engagement Skewness* means that the participants tend to engage towards the end of their workday. We used the skewness metric instead of simply looking at the engagement during the morning and the afternoon to account for individual differences in working hours. We found that 61.90% of the participants skewed towards engaging at the end of the day while 38.10% of the participants skewed towards the beginning of the day. Figure S7b shows the probability density distribution of intervention engagement per participant. We incorporated this skewness per participant in our analysis.

3.6 Passively Sensed Context

To understand the context surrounding a system-initiated intervention, we leveraged two sources of passively sensed data that required little or no action from the participants to collect: custom sensing software and Viva Insights.

The custom sensing software ran on participants' desktops to infer stress scores. Viva Insights captures de-identified activity aggregates in 30-minute windows for Microsoft tools across all devices associated with an individual's work account. While Viva Insights can obtain logs related to meetings, emails, chats, or calls across all devices with the same logged-in account, the sensing software can capture activities that may not be associated with work, such as browsing the internet or using non-Microsoft software. The smallest granularity of data aggregated by Viva Insights is 30-minutes, and the Viva Insights data stream can only be obtained retroactively. On the other hand, the sensing software can capture signals at every second or per event and in real-time. Because the sensing software or its components (i.e., camera) could be disabled by users or the user could be working on a different machine, there were gaps in the continuous streams of data. For example, 67.25% of the computer activity hours and 75.94% of the camera hours logged by the sensing software with engagement labels had less than 10 minutes of each hour represented. In comparison, only 5.42% of calendar hours and 3.30% of email hours had less than 10 minutes represented.

To evaluate the quality of our data sources, we conducted a correlation analysis to validate that the two data sources corroborate with each other where they overlap and to identify signals uniquely captured by each data source. All Pearson correlation coefficients between the two data sources

reported here are statistically significant ($p < 0.05$). We found that Viva Insights' *Chat Messages Count* is correlated with the sensing software's *Meeting App Keypress Count* and *Meeting App Mouse Move Count* (Pearson r=0.67 for both). Viva Insights' *Email Messages Sent* is correlated with the sensing software's *Mail App Keypress Count* and *Mail App Mouse Move Count* (Pearson r=0.32, 0.33, respectively). Similarly, Viva Insights' *Meeting Count* is correlated with the sensing software's *Calendar Meeting Count* (Pearson r=0.58). We found a strong correlation between the quality of the sensing software data and its count-based signals. For example, the fraction of the computer activity hours with data is correlated with *Number of Attention Signals* (Pearson r=0.82). We exclude computer activity and camera-based signals from the sensing software in the analysis due to its lack of temporal coverage (i.e., most of the hour has missing data). Because intervention nudges could be received through any device that Teams is installed, relying on Viva Insights data in areas where two data sources overlap is more reliable in obtaining a device-independent picture of the user's workplace context.

The sensing software also captured general user computer activity events such as mouse and keyboard interactions into a single metric, *Number of Attention Signals*, which could be an important indicator for presence. We hypothesized that presence at the computer could lead to higher engagement in interventions as the nudges were designed to grab the attention of participants at work. The range of values for *Number of Attention Signals* was fairly large (max=5705) compared to other variables, so we divided the values by 2000 to estimate a comparable coefficient and confidence intervals during modeling (i.e., to have odds ratios within 2 decimal points). When interpreting the effect sizes, we corrected for this factor of 2000. We also hypothesized that the likelihood of engaging in a stress-reduction intervention during active participation in a meeting is low. Therefore, we included *No Meeting Minutes* to represent the total number of minutes without a scheduled meeting with others and *Self Event Count* as the total number of calendar events with only the participant as the attendee.

From Viva Insights, we included *Meeting Counts*, *Adhoc Call Count*, and *Email Messages Sent/Read*. We excluded *Chat Messages Count* from our analysis because the nudges were delivered through Teams, and our data source cannot be used to discern if the messages were coming from the bot. Comparison of means revealed that there was a significant difference in *Chat Messages Count* between when participants were engaged and when they were not engaged in an intervention ($t(1312.6)=-3.452$, $p \ll 0.001$). Because Viva Insights data is limited to half-hourly windows, we associated the contextual metrics with each system-initiated intervention by taking the half-hour window that holds the nudge timestamp.

4 Additional Analyses

Below, we present ANOVA Type II and multicollinearity analysis results conducted for each logistic regression model. For each categorical variable with more than two levels, we also present the results of pairwise comparisons using Tukey's HSD procedure. All p-values for ANOVA analyses are adjusted by applying the Benjamini-Hochberg correction for multiple comparisons.

4.1 Predictors of *Engaged* model

Table S2: ANOVA Type II and Multicollinarity results for each predictor of *Engaged* model for all system-initiated nudges.

Predictor	Chisq	Df	P-value	Adj. p-value	VIF	VIF 95% CI
Age Group	5.83	2	0.054	0.155	1.46	[1.37, 1.56]
Gender	5.33	1	0.021	0.084	1.51	[1.42, 1.62]
Cognitive Reappraisal	4.47	1	0.034	0.115	1.55	[1.46, 1.67]
Expressive Suppression	2.46	1	0.117	0.234	1.48	[1.39, 1.58]
Resilience	0.26	1	0.608	0.680	2.23	[2.07, 2.41]
Agreeableness	2.06	1	0.151	0.252	1.92	[1.79, 2.07]
Conscientiousness	2.15	1	0.143	0.252	1.71	[1.60, 1.83]
Extraversion	0.18	1	0.667	0.680	1.60	[1.51, 1.72]
Neuroticism	0.17	1	0.680	0.680	2.08	[1.94, 2.25]
Openness	3.42	1	0.064	0.161	1.42	[1.34, 1.52]
Engagement Skewness	16.87	1	0.000	0.000	1.26	[1.20, 1.35]
Nudge Probability	2.73	1	0.098	0.219	1.10	[1.06, 1.18]
Meeting Counts	16.86	1	0.000	0.000	1.07	[1.03, 1.15]
No Meeting Minutes	0.32	1	0.569	0.680	1.04	[1.01, 1.15]
Self Event Counts	0.79	1	0.375	0.575	1.10	[1.06, 1.18]
Email Messages Sent	0.58	1	0.445	0.594	1.22	[1.16, 1.30]
Email Messages Read	0.70	1	0.403	0.575	1.18	[1.13, 1.26]
Adhoc Call Count	0.18	1	0.672	0.680	1.03	[1.01, 1.16]
Number of Attn. Signals	13.65	1	0.000	0.001	1.20	[1.14, 1.28]
Trigger Source	14.29	1	0.000	0.001	1.07	[1.04, 1.16]

Table S3: Pairwise comparisons of Age Group levels using Tukey's HSD for *Engaged* model.

Predictor	Estimate	Std. Error	z value	Pr(>|z|)
36-45 - 18-35	0.16	0.14	1.10	0.52
46+ - 18-35	0.39	0.16	2.40	0.04
46+ - 36-45	0.23	0.14	1.61	0.24

4.2 Predictors of *Distract Chosen* model

Table S4: ANOVA Type II and Multicollinarity results for each predictor of *Distract Chosen* model for all participant-chosen interventions.

Predictor	Chisq	Df	P-value	Adj. p-value	VIF	VIF 95% CI
Age Group	13.04	2	0.001	0.015	1.59	[1.45, 1.77]
Gender	4.15	1	0.042	0.208	1.60	[1.46, 1.79]
Cognitive Reappraisal	0.01	1	0.913	0.975	1.75	[1.59, 1.96]
Expressive Suppression	0.00	1	0.947	0.975	1.57	[1.43, 1.75]
Resilience	0.23	1	0.635	0.955	2.28	[2.05, 2.58]
Agreeableness	3.23	1	0.072	0.241	2.28	[2.04, 2.57]
Conscientiousness	3.29	1	0.070	0.241	1.88	[1.70, 2.11]
Extraversion	1.54	1	0.215	0.538	1.63	[1.49, 1.83]
Neuroticism	0.90	1	0.342	0.760	1.97	[1.78, 2.22]
Openness	16.34	1	0.000	0.001	1.31	[1.21, 1.45]
Engagement Skewness	0.28	1	0.599	0.955	1.38	[1.27, 1.54]
Nudge Probability	0.02	1	0.877	0.975	1.17	[1.09, 1.30]
Meeting Counts	0.74	1	0.390	0.780	1.06	[1.02, 1.24]
No Meeting Minutes	0.11	1	0.737	0.955	1.05	[1.01, 1.25]
Self Event Counts	0.09	1	0.764	0.955	1.15	[1.08, 1.28]
Email Messages Sent	0.16	1	0.686	0.955	1.28	[1.18, 1.42]
Email Messages Read	2.34	1	0.126	0.359	1.21	[1.13, 1.35]
Adhoc Call Count	0.09	1	0.762	0.955	1.06	[1.01, 1.24]
Number of Attn. Signals	8.58	1	0.003	0.023	1.23	[1.15, 1.37]
Trigger Source	0.00	1	0.975	0.975	1.10	[1.04, 1.25]

Table S5: Pairwise comparisons of Age Group levels using Tukey's HSD for *Distract Chosen* model.

Predictor	Estimate	Std. Error	z value	Pr(>|z|)
36-45 - 18-35	0.50	0.24	2.06	0.10
46+ - 18-35	-0.34	0.29	-1.16	0.47
46+ - 36-45	-0.84	0.25	-3.41	0.00

4.3 Predictors of *Calm Chosen* model

Table S6: ANOVA Type II and Multicollinarity results for each predictor of *Calm Chosen* model for all participant-chosen interventions.

Predictor	Chisq	Df	P-value	Adj. p-value	VIF	VIF 95% CI
Age Group	13.04	2	0.001	0.015	1.59	[1.45, 1.77]
Gender	4.15	1	0.042	0.208	1.60	[1.46, 1.79]
Cognitive Reappraisal	0.01	1	0.913	0.975	1.75	[1.59, 1.96]
Expressive Suppression	0.00	1	0.947	0.975	1.57	[1.43, 1.75]
Resilience	0.23	1	0.635	0.955	2.28	[2.05, 2.58]
Agreeableness	3.23	1	0.072	0.241	2.28	[2.04, 2.57]
Conscientiousness	3.29	1	0.070	0.241	1.88	[1.70, 2.11]
Extraversion	1.54	1	0.215	0.538	1.63	[1.49, 1.83]
Neuroticism	0.90	1	0.342	0.760	1.97	[1.78, 2.22]
Openness	16.34	1	0.000	0.001	1.31	[1.21, 1.45]
Engagement Skewness	0.28	1	0.599	0.955	1.38	[1.27, 1.54]
Nudge Probability	0.02	1	0.877	0.975	1.17	[1.09, 1.30]
Meeting Counts	0.74	1	0.390	0.780	1.06	[1.02, 1.24]
No Meeting Minutes	0.11	1	0.737	0.955	1.05	[1.01, 1.25]
Self Event Counts	0.09	1	0.764	0.955	1.15	[1.08, 1.28]
Email Messages Sent	0.16	1	0.686	0.955	1.28	[1.18, 1.42]
Email Messages Read	2.34	1	0.126	0.359	1.21	[1.13, 1.35]
Adhoc Call Count	0.09	1	0.762	0.955	1.06	[1.01, 1.24]
Number of Attn. Signals	8.58	1	0.003	0.023	1.23	[1.15, 1.37]
Trigger Source	0.00	1	0.975	0.975	1.10	[1.04, 1.25]

Table S7: Pairwise comparisons of Age Group levels using Tukey's HSD for *Calm Chosen* model.

Predictor	Estimate	Std. Error	z value	Pr(>|z|)
36-45 - 18-35	-0.47	0.22	-2.13	0.08
46+ - 18-35	-0.05	0.26	-0.19	0.98
46+ - 36-45	0.42	0.21	1.97	0.12

4.4 Predictors of *Address Chosen* model

Table S8: ANOVA Type II and Multicollinarity results for each predictor of *Address Chosen* model for all participant-chosen interventions.

Predictor	Chisq	Df	P-value	Adj. p-value	VIF	VIF 95% CI
Age Group	4.53	2	0.104	0.301	1.85	[1.67, 2.07]
Gender	0.76	1	0.383	0.589	1.74	[1.57, 1.94]
Cognitive Reappraisal	11.46	1	0.001	0.014	1.68	[1.53, 1.88]
Expressive Suppression	0.21	1	0.649	0.722	1.95	[1.75, 2.19]
Resilience	3.85	1	0.050	0.297	2.09	[1.88, 2.35]
Agreeableness	3.59	1	0.058	0.297	2.05	[1.84, 2.30]
Conscientiousness	2.84	1	0.092	0.301	2.12	[1.91, 2.39]
Extraversion	1.87	1	0.172	0.381	1.51	[1.38, 1.68]
Neuroticism	3.55	1	0.059	0.297	2.21	[1.98, 2.49]
Openness	1.38	1	0.241	0.438	1.57	[1.44, 1.76]
Engagement Skewness	0.38	1	0.538	0.722	1.32	[1.22, 1.46]
Nudge Probability	0.87	1	0.350	0.584	1.11	[1.05, 1.25]
Meeting Counts	0.29	1	0.591	0.722	1.06	[1.01, 1.24]
No Meeting Minutes	0.02	1	0.893	0.940	1.06	[1.02, 1.24]
Self Event Counts	0.34	1	0.561	0.722	1.21	[1.12, 1.34]
Email Messages Sent	0.00	1	0.945	0.945	1.34	[1.24, 1.49]
Email Messages Read	2.41	1	0.120	0.301	1.20	[1.12, 1.33]
Adhoc Call Count	0.23	1	0.631	0.722	1.05	[1.01, 1.26]
Number of Attn. Signals	2.62	1	0.106	0.301	1.22	[1.13, 1.36]
Trigger Source	1.42	1	0.234	0.438	1.15	[1.08, 1.29]

Table S9: Pairwise comparisons of Age Group levels using Tukey's HSD for *Address Chosen* model.

Predictor	Estimate	Std. Error	z value	Pr(>|z|)
36-45 - 18-35	-0.13	0.32	-0.41	0.91
46+ - 18-35	0.47	0.33	1.43	0.32
46+ - 36-45	0.60	0.29	2.06	0.10

4.5 Predictors of *Engaged after chosen* model

Table S10: ANOVA Type II and Multicollinarity results for each predictor of *Engaged after chosen* model for all interventions after participants chose a category.

Predictor	Chisq	Df	P-value	Adj. p-value	VIF	VIF 95% CI
Age Group	1.80	2	0.407	0.520	2.17	[1.95, 2.44]
Gender	2.44	1	0.118	0.290	1.64	[1.49, 1.83]
Cognitive Reappraisal	0.54	1	0.464	0.561	1.97	[1.77, 2.21]
Expressive Suppression	2.59	1	0.108	0.290	2.14	[1.92, 2.40]
Resilience	4.82	1	0.028	0.215	2.76	[2.46, 3.12]
Agreeableness	0.80	1	0.370	0.520	2.67	[2.38, 3.02]
Conscientiousness	1.24	1	0.265	0.436	1.87	[1.69, 2.10]
Extraversion	2.56	1	0.110	0.290	1.80	[1.63, 2.02]
Neuroticism	2.99	1	0.084	0.290	2.59	[2.31, 2.93]
Openness	1.47	1	0.225	0.431	1.36	[1.25, 1.51]
Engagement Skewness	0.00	1	0.957	0.957	1.34	[1.24, 1.49]
Nudge Probability	0.08	1	0.773	0.847	1.17	[1.09, 1.30]
Meeting Counts	2.96	1	0.086	0.290	1.11	[1.05, 1.25]
No Meeting Minutes	0.04	1	0.841	0.879	1.08	[1.03, 1.23]
Self Event Counts	2.45	1	0.117	0.290	1.11	[1.05, 1.24]
Email Messages Sent	0.76	1	0.384	0.520	1.20	[1.12, 1.34]
Email Messages Read	1.02	1	0.312	0.478	1.19	[1.11, 1.32]
Adhoc Call Count	1.86	1	0.173	0.361	1.14	[1.07, 1.27]
Number of Attn. Signals	0.28	1	0.597	0.686	1.24	[1.15, 1.38]
Trigger Source	2.34	1	0.126	0.290	1.14	[1.07, 1.28]
Category	2.75	2	0.253	0.436	2.63	[2.35, 2.98]
Modality	8.24	2	0.016	0.192	2.26	[2.03, 2.55]
Location	8.18	2	0.017	0.192	1.41	[1.30, 1.57]

Table S11: Pairwise comparisons of Age Group levels using Tukey's HSD for *Engaged after chosen* model.

Predictor	Estimate	Std. Error	z value	Pr(>|z|)
36-45 - 18-35	0.05	0.34	0.16	0.99
46+ - 18-35	0.50	0.44	1.14	0.48
46+ - 36-45	0.45	0.37	1.23	0.43

Table S12: Pairwise comparisons of Category levels using Tukey's HSD for *Engaged after chosen* model.

Predictor	Estimate	Std. Error	z value	Pr(>|z|)
Distract - Address	0.60	0.48	1.26	0.41
Calm - Address	0.73	0.43	1.71	0.20
Calm - Distract	0.13	0.32	0.41	0.91

Table S13: Pairwise comparisons of Modality levels using Tukey's HSD for *Engaged after chosen* model.

Predictor	Estimate	Std. Error	z value	Pr(>|z|)
prompt - conversation	1.26	0.50	2.54	0.03
video - conversation	1.77	0.65	2.73	0.02
video - prompt	0.51	0.41	1.23	0.42

Table S14: Pairwise comparisons of Location levels using Tukey's HSD for *Engaged after chosen* model.

Predictor	Estimate	Std. Error	z value	Pr(>|z|)
Inside - At desk	-0.84	0.33	-2.56	0.03
Outside - At desk	-1.07	0.59	-1.82	0.15
Outside - Inside	-0.24	0.62	-0.38	0.92

4.6 Predictors of *Liked* model

Table S15: ANOVA Type II and Multicollinearity results for each predictor of *Liked* model for all interventions that participants engaged in.

Predictor	Chisq	Df	P-value	Adj. p-value	VIF	VIF 95% CI
Age Group	9.89	2.00	0.007	0.038	1.77	[1.59, 2.01]
Gender	12.79	1.00	0.000	0.004	1.75	[1.58, 1.99]
Cognitive Reappraisal	6.83	1.00	0.009	0.038	1.81	[1.62, 2.05]
Expressive Suppression	4.06	1.00	0.044	0.151	1.77	[1.59, 2.00]
Resilience	0.31	1.00	0.575	0.767	2.31	[2.04, 2.63]
Agreeableness	3.49	1.00	0.062	0.185	2.26	[2.00, 2.58]
Conscientiousness	2.06	1.00	0.152	0.308	2.00	[1.79, 2.27]
Extraversion	6.75	1.00	0.009	0.038	1.66	[1.50, 1.87]
Neuroticism	1.49	1.00	0.222	0.381	2.02	[1.80, 2.29]
Openness	0.01	1.00	0.937	1.000	1.50	[1.36, 1.69]
Engagement Skewness	2.03	1.00	0.154	0.308	1.32	[1.22, 1.49]
Nudge Probability	6.89	1.00	0.009	0.038	1.18	[1.10, 1.33]
Meeting Counts	0.11	1.00	0.743	0.869	1.08	[1.02, 1.26]
No Meeting Minutes	0.09	1.00	0.761	0.869	1.07	[1.02, 1.26]
Self Event Counts	2.60	1.00	0.107	0.257	1.21	[1.13, 1.37]
Email Messages Sent	0.00	1.00	0.965	1.000	1.31	[1.21, 1.48]
Email Messages Read	0.00	1.00	1.000	1.000	1.26	[1.16, 1.42]
Adhoc Call Count	0.72	1.00	0.395	0.557	1.07	[1.02, 1.26]
Number of Attn. Signals	0.80	1.00	0.370	0.554	1.30	[1.20, 1.46]
Trigger Source	0.93	1.00	0.335	0.536	1.17	[1.09, 1.32]
Category	0.93	2.00	0.627	0.792	2.08	[1.85, 2.37]
Stress Reduction	19.51	1.00	0.000	0.000	1.14	[1.06, 1.29]
Modality	3.19	2.00	0.203	0.375	1.87	[1.68, 2.13]
Location	4.61	2.00	0.100	0.257	1.17	[1.09, 1.32]

Table S16: Pairwise comparisons of Age Group levels using Tukey's HSD for *Liked* model.

Predictor	Estimate	Std. Error	z value	Pr(>|z|)
36-45 - 18-35	-0.67	0.27	-2.52	0.03
46+ - 18-35	0.01	0.31	0.03	1.00
46+ - 36-45	0.68	0.26	2.58	0.03

Table S17: Pairwise comparisons of Category levels using Tukey's HSD for *Liked* model.

Predictor	Estimate	Std. Error	z value	Pr(>|z|)
Calm - Distract	-0.23	0.25	-0.92	0.62
Address - Distract	-0.06	0.38	-0.16	0.99
Address - Calm	0.16	0.35	0.47	0.88

Table S18: Pairwise comparisons of Modality levels using Tukey's HSD for *Liked* model.

Predictor	Estimate	Std. Error	z value	Pr(>|z|)
prompt - conversation	-0.65	0.55	-1.17	0.45
video - conversation	-0.29	0.61	-0.47	0.88
video - prompt	0.35	0.27	1.32	0.36

Table S19: Pairwise comparisons of Location levels using Tukey's HSD for *Liked* model.

Predictor	Estimate	Std. Error	z value	Pr(>|z|)
Inside - At desk	-0.37	0.28	-1.34	0.36
Outside - At desk	0.94	0.62	1.53	0.26
Outside - Inside	1.31	0.66	1.99	0.10

4.7 Predictors of *Improved* model

Table S20: ANOVA Type II and Multicollinarity results for each predictor of *Improved* model for all interventions that participants engaged in.

Predictor	Chisq	Df	P-value	Adj. p-value	VIF	VIF 95% CI
Age Group	3.78	2.00	0.151	0.299	1.69	[1.52, 1.91]
Gender	7.77	1.00	0.005	0.027	1.88	[1.68, 2.13]
Cognitive Reappraisal	6.74	1.00	0.009	0.039	2.03	[1.81, 2.31]
Expressive Suppression	0.01	1.00	0.938	0.938	2.10	[1.87, 2.39]
Resilience	0.75	1.00	0.388	0.606	2.47	[2.18, 2.82]
Agreeableness	5.68	1.00	0.017	0.054	2.63	[2.32, 3.01]
Conscientiousness	1.40	1.00	0.237	0.424	2.06	[1.83, 2.34]
Extraversion	0.36	1.00	0.549	0.695	1.77	[1.59, 2.01]
Neuroticism	9.57	1.00	0.002	0.014	2.54	[2.25, 2.91]
Openness	0.27	1.00	0.606	0.721	1.38	[1.26, 1.56]
Engagement Skewness	0.35	1.00	0.556	0.695	1.38	[1.26, 1.55]
Nudge Probability	9.38	1.00	0.002	0.014	1.25	[1.16, 1.41]
Meeting Counts	0.39	1.00	0.535	0.695	1.11	[1.04, 1.27]
No Meeting Minutes	0.92	1.00	0.337	0.561	1.14	[1.06, 1.29]
Self Event Counts	0.65	1.00	0.420	0.617	1.17	[1.09, 1.32]
Email Messages Sent	0.08	1.00	0.777	0.883	1.32	[1.21, 1.48]
Email Messages Read	2.63	1.00	0.105	0.238	1.22	[1.13, 1.38]
Adhoc Call Count	0.01	1.00	0.913	0.938	1.06	[1.01, 1.27]
Number of Attn. Signals	2.92	1.00	0.087	0.218	1.27	[1.17, 1.43]
Trigger Source	0.05	1.00	0.820	0.891	1.25	[1.16, 1.41]
Category	8.29	2.00	0.016	0.054	1.97	[1.76, 2.24]
Stress Before	116.37	1.00	0.000	0.000	2.14	[1.91, 2.44]
Rating	41.00	1.00	0.000	0.000	1.40	[1.28, 1.57]
Modality	6.93	2.00	0.031	0.087	1.68	[1.52, 1.90]
Location	3.72	2.00	0.155	0.299	1.19	[1.11, 1.34]

Table S21: Pairwise comparisons of Age Group levels using Tukey's HSD for *Improved* model.

Predictor	Estimate	Std. Error	z value	Pr(>|z|)
36-45 - 18-35	-0.54	0.34	-1.59	0.25
46+ - 18-35	-0.02	0.36	-0.05	1.00
46+ - 36-45	0.52	0.32	1.64	0.23

Table S22: Pairwise comparisons of Category levels using Tukey's HSD for *Improved* model.

Predictor	Estimate	Std. Error	z value	Pr(>|z|)
Calm - Distract	-0.85	0.31	-2.77	0.02
Address - Distract	-0.91	0.46	-2.00	0.11
Address - Calm	-0.07	0.40	-0.16	0.98

Table S23: Pairwise comparisons of Modality levels using Tukey's HSD for *Improved* model.

Predictor	Estimate	Std. Error	z value	Pr(>|z|)
prompt - conversation	1.90	0.80	2.37	0.04
video - conversation	1.73	0.86	2.00	0.10
video - prompt	-0.17	0.32	-0.52	0.85

Table S24: Pairwise comparisons of Location levels using Tukey's HSD for *Improved* model.

Predictor	Estimate	Std. Error	z value	Pr(>|z|)
Inside - At desk	0.16	0.34	0.46	0.88
Outside - At desk	-1.47	0.88	-1.67	0.20
Outside - Inside	-1.63	0.92	-1.76	0.17

References

[1] Microsoft Learn. Overview of Microsoft Graph. https://learn.microsoft.com/en-us/graph/overview, 2023. (Accessed 2024-05-25).

[2] Microsoft Learn. Advanced Insights Metrics Description. https://learn.microsoft.com/en-us/previous-versions/viva/insights/use/metric-definitions, 2023. (Accessed 2024-05-25).

[3] Xin Liu, Josh Fromm, Shwetak Patel, and Daniel McDuff. Multi-task temporal shift attention networks for on-device contactless vitals measurement. *NeurIPS*, 2020.

[4] Microsoft. Microsoft Bot Framework. https://dev.botframework.com, 2024. (Accessed 2024-05-25).

[5] Microsoft. Adaptive Cards. https://adaptivecards.io, 2024. (Accessed 2024-05-25).

[6] Akane Sano, Paul Johns, and Mary Czerwinski. Designing opportune stress intervention delivery timing using multi-modal data. In *2017 Seventh International Conference on Affective Computing and Intelligent Interaction (ACII)*, pages 346–353. IEEE, 2017.

[7] Gloria Mark, Yiran Wang, and Melissa Niiya. Stress and multitasking in everyday college life: an empirical study of online activity. In *Proceedings of the SIGCHI conference on human factors in computing systems*, pages 41–50, 2014.

[8] Gloria Mark, Shamsi T Iqbal, Mary Czerwinski, Paul Johns, Akane Sano, and Yuliya Lutchyn. Email duration, batching and self-interruption: Patterns of email use on productivity and stress. In *Proceedings of the 2016 CHI conference on human factors in computing systems*, pages 1717–1728. ACM, 2016.

[9] Gloria Mark, Shamsi Iqbal, and Mary Czerwinski. How blocking distractions affects workplace focus and productivity. In *Proceedings of the 2017 ACM International Joint Conference on Pervasive and Ubiquitous Computing and Proceedings of the 2017 ACM International Symposium on Wearable Computers*, pages 928–934, 2017.

[10] Daniel McDuff, Eunice Jun, Kael Rowan, and Mary Czerwinski. Longitudinal observational evidence of the impact of emotion regulation strategies on affective expression. *IEEE Transactions on Affective Computing*, 2019.

[11] Rosenberg Ekman. *What the face reveals: Basic and applied studies of spontaneous expression using the Facial Action Coding System (FACS)*. Oxford University Press, USA, 1997.

[12] Leah M Mayo and Markus Heilig. In the face of stress: Interpreting individual differences in stress-induced facial expressions. *Neurobiology of stress*, 10:100166, 2019.

[13] Karim Sadik Kassam. *Assessment of emotional experience through facial expression*. Harvard University, 2010.

[14] Tanja GM Vrijkotte, Lorenz JP Van Doornen, and Eco JC De Geus. Effects of work stress on ambulatory blood pressure, heart rate, and heart rate variability. *Hypertension*, 35(4):880–886, 2000.

[15] Nis Hjortskov, Dag Rissén, Anne Katrine Blangsted, Nils Fallentin, Ulf Lundberg, and Karen Søgaard. The effect of mental stress on heart rate variability and blood pressure during computer work. *European journal of applied physiology*, 92(1):84–89, 2004.

[16] Joshua M Smyth and Kristin E Heron. Is providing mobile interventions "just-in-time" helpful? an experimental proof of concept study of just-in-time intervention for stress management. In *2016 IEEE Wireless Health (WH)*, pages 1–7. IEEE, 2016.

[17] Predrag Klasnja, Beverly L Harrison, Louis LeGrand, Anthony LaMarca, Jon Froehlich, and Scott E Hudson. Using wearable sensors and real time inference to understand human recall of routine activities. In *Proceedings of the 10th international conference on Ubiquitous computing*, pages 154–163, 2008.

[18] Andrew C Butler, Jason E Chapman, Evan M Forman, and Aaron T Beck. The empirical status of cognitive-behavioral therapy: a review of meta-analyses. *Clinical psychology review*, 26(1):17–31, 2006.

[19] Anita Lungu and Marsha M Linehan. Dialectical behavior therapy: A comprehensive multi-and transdiagnostic intervention. *The Oxford handbook of cognitive and behavioral therapies*, pages 200–214, 2016.

[20] Marsha Linehan. *DBT Skills training manual*. Guilford Publications, 2014.

[21] Eric Horvitz. Principles of mixed-initiative user interfaces. In *Proceedings of the SIGCHI conference on Human Factors in Computing Systems*, pages 159–166, 1999.

[22] Jessica Schroeder, Jina Suh, Chelsey Wilks, Mary Czerwinski, Sean A Munson, James Fogarty, and Tim Althoff. Data-driven implications for translating evidence-based psychotherapies into technology-delivered interventions. In *Proceedings of the 14th EAI International Conference on Pervasive Computing Technologies for Healthcare*, pages 274–287, 2020.

[23] Mina Westman and Dalia Etzion. The impact of vacation and job stress on burnout and absenteeism. *Psychology & Health*, 16(5):595–606, 2001.

[24] Esther Howe, Jina Suh, Mehrab Bin Morshed, Daniel McDuff, Kael Rowan, Javier Hernandez, Marah Ihab Abdin, Gonzalo Ramos, Tracy Tran, and Mary P Czerwinski. Design of digital workplace stress-reduction intervention systems: Effects of intervention type and timing. In *CHI Conference on Human Factors in Computing Systems*, pages 1–16, 2022.
